# Amphiregulin shapes mitochondrial delivery and immune modulation by microvescicles

**DOI:** 10.1016/j.isci.2026.117339

**Published:** 2026-09-03

**Authors:** Giuseppe Prencipe, Adrián Cerveró-Varona, Alessia Peserico, Angelo Canciello, Verdiana Di Giulio, Hélder A. Santos, Antti Tuhkala, Chika Takano, Toshio Miki, Valentina Russo, Maura Turriani, Oriana Di Giacinto, Barbara Barboni

**Affiliations:** 1 Department of Biosciences and Agro-Food and Environmental Technologies, University of Teramo, 64100 Teramo, Italy; 2 Department of Biomaterials and Biomedical Technology, The Personalized Medicine Research Institute (PRECISION), University Medical Center Groningen, University of Groningen, Groningen, the Netherlands; 3 Institute of Biotechnology, University of Helsinki, Finland; 4 Division of Microbiology, Department of Pathology and Microbiology, Nihon University School of Medicine, Tokyo, Japan; 5 Department of Pediatrics and Child Health, Nihon University School of Medicine, Tokyo, Japan; 6 Department of Physiology, Nihon University School of Medicine, Tokyo, Japan

**Keywords:** amniotic epithelial cells, extracellular vesicles, LPS, amphiregulin, cell-free therapy, mitochondrial transfer, immune response, immunotherapy

## Abstract

Amniotic epithelial cells (AECs) exert potent paracrine immunoregulatory functions, yet whether inflammatory cues enhance the biological activity of their extracellular vesicles (EVs) remains unclear. To address this question, we characterized the secretome of ovine AECs under basal and lipopolysaccharide (LPS)-stimulated conditions using label-free proteomics and genetic approaches. Inflammatory stimulation profoundly remodeled the microvesicles (MVs) cargo, enriching 101 proteins associated with metabolic and regulatory pathways. Among the top 5% were two immune-related proteins, including amphiregulin (AREG), an epidermal growth factor receptor ligand involved in immune regulation and tissue remodeling. AREG silencing reduced AREG-positive MVs and was associated with altered mitochondrial cargo content and transfer to immune cells. Consequently, siAREG-MVs showed reduced uptake by PBMCs and T cells, resulting in diminished suppression of proliferation and CD3-dependent NFAT activation. Exogenous AREG partially restored these functions. Collectively, these findings identify inflammation-driven AREG signaling as a key mechanism enhancing the immunomodulatory potential of AEC-derived MVs for cell-free immunotherapy.

## Highlights

• LPS remodels the AEC-derived MV proteome, enriching 101 proteins including AREG
• AREG silencing reduces mitochondrial cargo in AEC-derived MVs
• AREG silencing impairs MV-mediated mitochondrial transfer to immune cells
• MVs from AREG-silenced AECs fail to suppress PBMC proliferation and NFAT activation

## Graphical abstract

**Figure:**
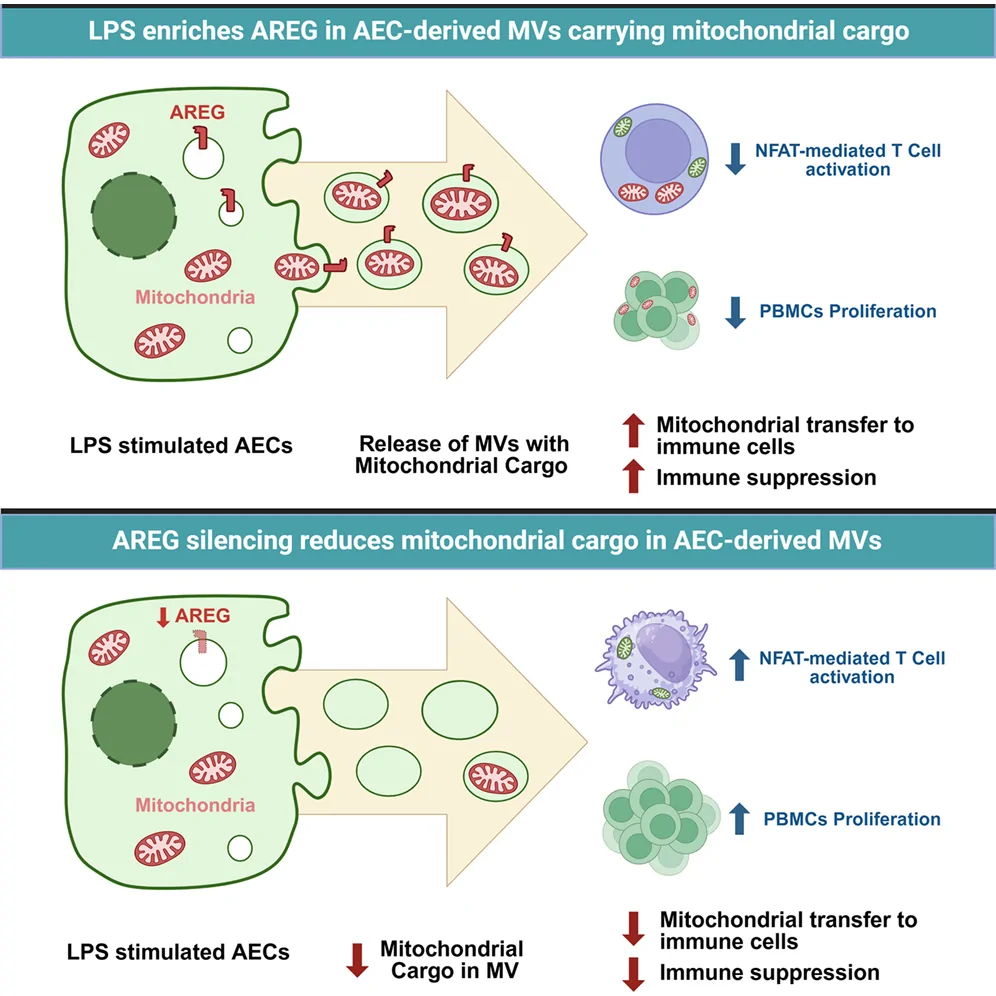

## Teaser

Biological sciences; Immunology; Cell biology

## Introduction

The immune response to tissue damage plays a pivotal role in early recovery by orchestrating cellular repair mechanisms that restore homeostasis. Its success depends on precise spatiotemporal regulation; disruptions in this balance can lead to chronic inflammation, fibrosis, or autoimmunity.^1^^,^^2^^,^^3^ Such dysregulations represent a major barrier to regenerative outcomes, underscoring the need for a deeper understanding of immune-tissue interactions in health and disease.^4^^,^^5^

In this context, researchers have sought therapeutic strategies to effectively modulate immune responses.^4^^,^^6^^,^^7^^,^^8^ Amniotic epithelial cells (AECs) have emerged as promising candidates due to their stem-like plasticity and potent immunoregulatory functions.^6^^,^^9^^,^^10^^,^^11^^,^^12^^,^^13^^,^^14^^,^^15^^,^^16^^,^^17^ Derived from the innermost placental layer, AECs combine embryonic and adult stem cell traits and secrete immunomodulatory molecules that promote tissue repair.^9^^,^^10^^,^^11^^,^^13^^,^^18^^,^^19^^,^^20^^,^^21^

AEC-conditioned media (AEC-CM) broadly suppresses immune cell activation through multiple mechanisms, including the suppression of peripheral blood mononuclear cells (PBMCs) proliferation,^15^^,^^16^^,^^19^^,^^22^^,^^23^^,^^24^ the regulation of antigen-presenting cell recruitment, maturation, and differentiation, particularly of macrophages and dendritic cells,^19^^,^^25^^,^^26^^,^^27^ and the modulation of T cell activation.^15^^,^^16^^,^^24^^,^^28^ This cell-free system serves as a therapeutic tool, delivering a complex cargo of bioactive molecules, including growth factors, cytokines, and extracellular vesicles (EVs), that influence immune cell behavior.^16^^,^^22^^,^^28^^,^^29^^,^^30^^,^^31^^,^^32^

Within this network, amphiregulin (AREG) has emerged as a key effector that modulates both tissue repair and immune regulation.^16^^,^^33^^,^^34^^,^^35^^,^^36^ Recent studies demonstrate that AREG secretion by AEC is controlled by the COX-2/PGE2/EP4 axis and that AREG contributes to immune suppression via TGF-β signaling.^16^^,^^33^ Functionally, AREG suppresses PBMC activation and inhibits NFAT signaling in T cells, in a feedback-regulated manner triggered by inflammatory stimuli like lipopolysaccharide (LPS).^16^^,^^37^

Alongside AREG, AEC-derived microvesicles (AEC_MV_), a subclass of EVs (100–1,000 nm in diameter), also contribute to AEC-driven paracrine immunomodulation by transferring organelle-rich bioactive cargo, including functional mitochondria, lipids, and endoplasmic reticulum.^15^^,^^30^^,^^38^ Upon internalization via macropinocytosis,^15^^,^^39^ AEC_MV_ induce mitochondrial hyperpolarization and caspase-3 activation in PBMCs and Jurkat cells, ultimately suppressing immune activation and promoting immune homeostasis.^15^

While the independent immunomodulatory roles of AREG^16^^,^^18^^,^^33^^,^^40^ and AEC_MV_^16^ are well-documented, whether AREG contributes functionally as a component of AEC_MV_ remains unknown. Using a proteomic approach, we found that AREG is selectively incorporated into AEC_MV_ and that its abundance increases following LPS stimulation. By silencing AREG in AECs and validating knockdown at both the mRNA and protein levels, we dissected its role using a dual translational model in which ovine MVs were tested on allogeneic ovine PBMCs and human Jurkat cells. MVs from AREG-silenced AECs displayed impaired immunosuppressive activity, failing to effectively inhibit PBMC proliferation and NFAT activation in Jurkat cells. Moreover, mitochondrial transfer from MVs to immune cells was significantly reduced upon AREG depletion.

These findings identify AREG as a critical cargo of AEC_MV_, required for their immunoregulatory capacity and potentially involved in mediating MV cargo targeting recipient immune cells. They uncover a previously unrecognized mechanistic axis with therapeutic relevance for cell-free treatment of immune-mediated inflammation and potentially broader regenerative applications.

## Results

### Distinct proteomic signature and inflammatory responsiveness define AEC_MV_

The study rationale stems from our prior lipidomic profiling, which revealed a selective enrichment in signaling lipids and organelle-associated species, particularly those linked to mitochondria and the endoplasmic reticulum, suggesting the presence of coordinated functional complexes.^15^

While these findings pointed to a structural and bioactive specialization of MV derived from AECs, they raised the question of which protein components might orchestrate or synergize with the lipid-based effects, ultimately mediating immune cell modulation. Thus, a proteomic approach was undertaken to gain deeper insights into the molecular effectors embedded within the MV cargo and how their composition reflects the cell’s activation state.

**Figure 1:**
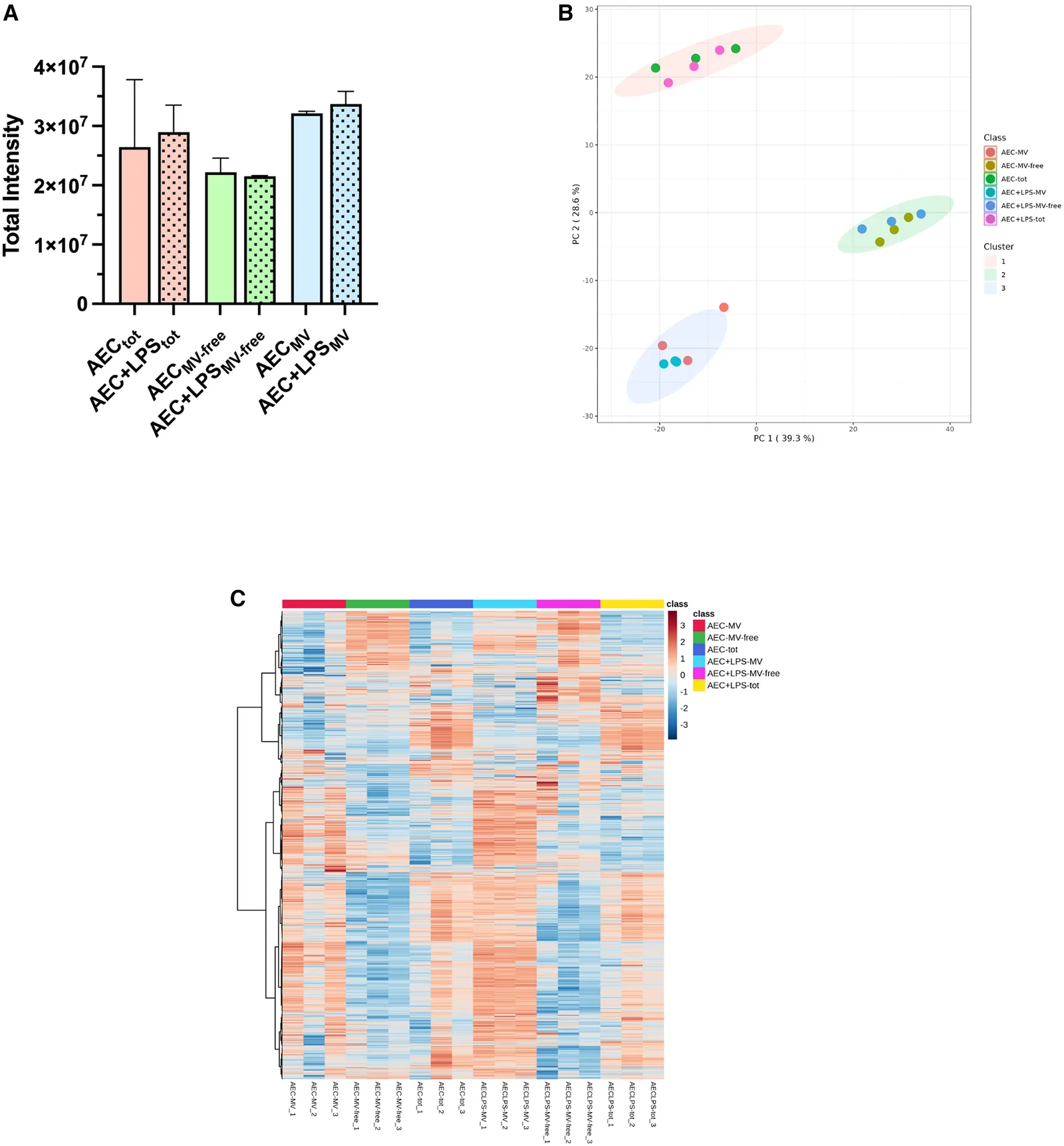
Distinct proteomic signature and inflammatory responsiveness define AEC_MV_ (A) Total protein signal intensities obtained via label-free mass spectrometry across the three secretome fractions: AEC ± LPS_tot_, AEC ± LPS_MV-free_, and AEC ± LPS_MV_. Data are presented as mean ± SD from three biological independent experiments. Statistical comparisons were performed using one-way ANOVA followed by Tukey’s multiple-comparisons test; no significant differences were detected among the experimental groups. (B) Bidimensional PCA was used for dimensionality reduction and discrimination between experimental groups. Each point represents an individual biological replicate. (C) Hierarchical heatmap based on Pearson’s correlation coefficients illustrates the proteomic signature across all secretome fractions. Data normality was assessed using the D’Agostino-Pearson’s test. Correlations between variables were subsequently evaluated using Pearson’s correlation coefficient.

An mass spectrometry (MS)-based proteomic analysis was conducted on MV-enriched fraction (AEC_MV_) and compared to that of total conditioned medium (AEC_tot_) and MV-depleted fraction (AEC_MV-free_). According to our previous studies,^15^^,^^16^ each fraction was obtained from AECs in a native state and following stimulation with LPS to mimic inflammatory conditions. We generated a proteomic dataset comprising 2,456 proteins referenced against the *Ovis aries* UniProt database (Table S1). Protein quantification was based on Data-Independent Acquisition by Neural Networks (DIA-NN)-derived normalized protein-group intensity values. These values reflect the relative abundance of proteins based on ion signal intensity and were utilized for intra-experimental comparisons. Proteomic profiling revealed comparable levels of total protein signal intensity across the different secretome fractions, with no significant variation observed regardless of the inflammatory status of the cells (Figure 1A). This indicates that the overall protein content remains consistent among AEC_tot_, AEC_MV_, and AEC_MV-free_ fractions.

To probe qualitative differences among the secretome fractions, we employed dimensionality reduction via principal component analysis (PCA) (Figure 1B) and hierarchical clustering presented as a heatmap (Figure 1C). The PCA revealed that 68% of the overall variance could be explained by the first two principal components, effectively distinguishing three discrete clusters corresponding to the different secretome fractions (AEC ± LPS_tot_, AEC ± LPS_MV-free_, and AEC ± LPS_MV_). This indicates that despite comparable total protein loads, the fractions differ markedly in protein composition. Consistently, the heatmap highlighted distinct abundance patterns: several protein subsets were specifically enriched in the AEC ± LPS_MV_ fraction, whereas AEC_tot_ and AEC_MV-free_ shared a different profile (Figure 1C). Finally, a pairwise correlation matrix (Figure S1) confirmed high internal consistency within each experimental group, particularly for AEC ± LPS_MV_, while demonstrating clear divergence between the three fractions.

### LPS stimulation remodels the immune-related proteomic cargo of AEC_MV_

**Figure 2:**
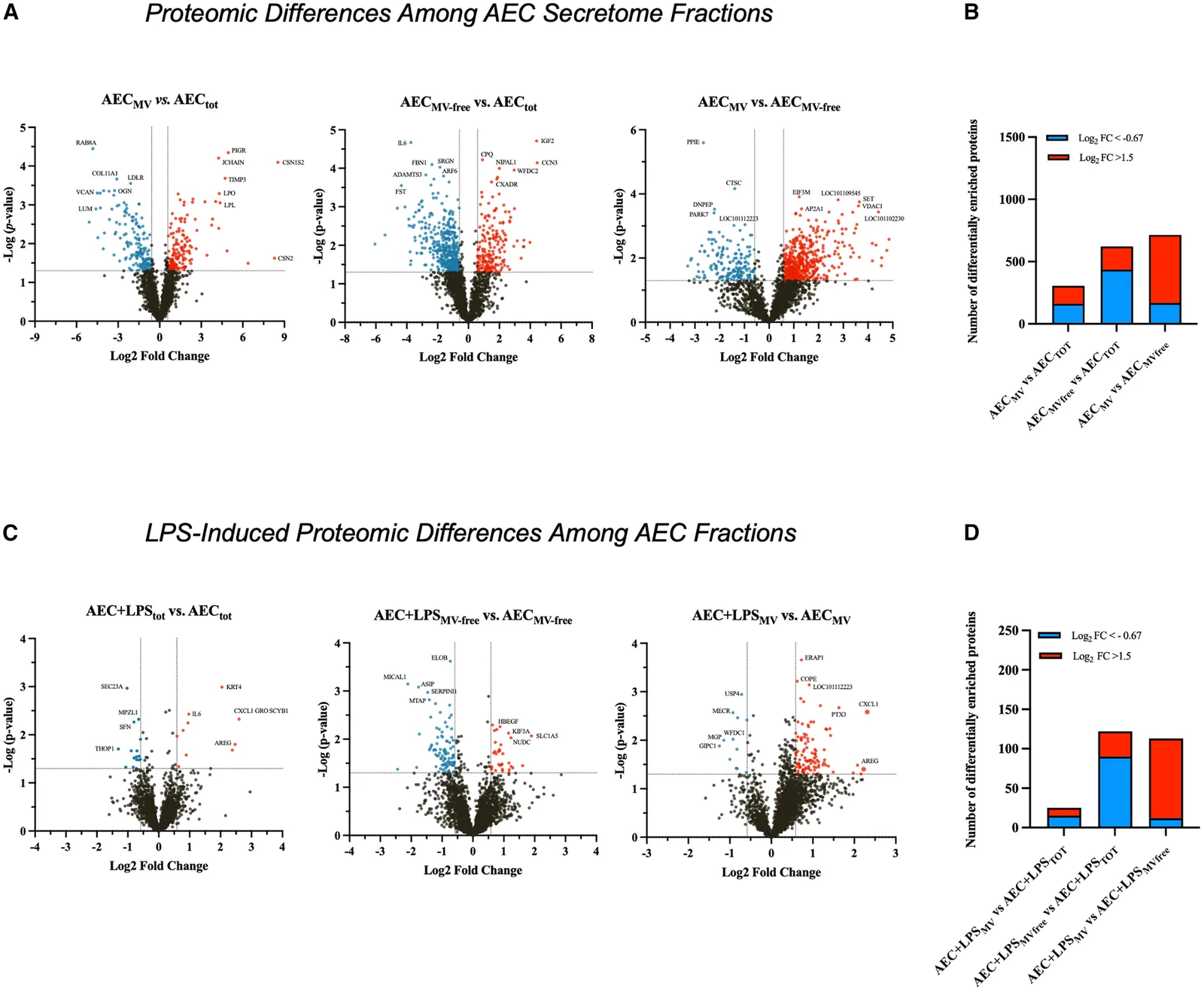
AEC_MV_ undergo proteomic cargo remodeling in response to LPS stimulation (A) Volcano plots showing differentially abundant proteins under basal conditions in AEC_MV_ compared with the other secretome fractions, AEC_tot_ and AEC_MV-free_. (B) Number of proteins displaying increased (log_2_FC > +1.5) or decreased (log_2_FC < −0.67) abundance in the indicated basal inter-fraction comparisons. (C) Volcano plots showing LPS-induced changes in protein abundance within each secretome fraction. (D) Number of proteins displaying increased (log_2_FC > +1.5) or decreased (log_2_FC < −0.67) abundance following LPS stimulation, highlighting the predominant response of the AEC_MV_ fraction. For each pairwise comparison, *p* values were calculated using Student’s *t* test implemented in the Python SciPy package and adjusted for multiple comparisons using the Benjamini-Hochberg method implemented in the statsmodels package. Volcano plots were generated using GraphPad prism. Proteins with a Benjamini-Hochberg-adjusted *p* < 0.05 and log_2_FC > +1.5 or log_2_FC < −0.67 were classified as proteins with increased or decreased abundance, respectively. Data were obtained from three biological independent experiments.

Volcano plot analyses were used to dissect both basal inter-fraction differences and LPS-induced changes (Figure 2). Volcano plot analyses applying a significance threshold of *p* < 0.05 combined with a Log2 fold change (FC) cutoff of +1.5 for proteins showing increasing abundance and −0.67 for proteins showing decreased abundance, revealed a limited proteomic divergence between the MV and MV-free fractions (Figure 2A), yielding the lowest number of differentially enriched proteins among all pairwise comparisons (144 proteins enriched and 162 depleted) (Figure 2B).

In contrast, the MV-free fraction demonstrated a substantial number of downregulated proteins compared to the total fraction (435 proteins enriched versus 210 depleted) (Figure 2B). Kyoto Encyclopedia of Genes and Genomes (KEGG) pathways indicated that these lower abundance proteins were mainly associated with pathways involved in protein processing (ribosome) and mitochondrial metabolism (carbon metabolism, trichloroacetic acid [TCA] cycle) and reply to environmental signals (PI3K-Akt and HIF1alpha signaling) (Tables 1 and S2). Conversely, higher abundance proteins in the MV-free fraction were associated with cellular processes and metabolic degradation pathways (lysosome, glycosaminoglycan, and valine, leucine, isoleucine degradation pathways) (Tables 1 and S2).

**Table 1:** Distinct KEGG pathway classification of differentially abundant proteins

Fraction	Metabolism	Genetic information Processing	Environmental information Processing	Cellular processes	Organismal/immune system	Diseases
**AEC**_**MV**_**vs. AEC**_**TOT**_	**oxidative phosphorylation**	**proteasome; ribosome**	PI3K-Akt signaling pathway; ECM-receptor interaction	focal adhesion; AGE–RAGE signaling pathway	complement and coagulation cascades; protein digestion and absorption; relaxin signaling pathway	human papillomavirus infection; amoebiasis; pathways in cancer **Huntington disease; Alzheimer disease; Prion disease; Parkinson disease; amyotrophic lateral sclerosis; proteoglycans in cancer**
**AEC**_**MV-free**_**vs. AEC**_**TOT**_	TCA cycle; metabolic pathways *carbon metabolism* **glycosaminoglycan degradation; cysteine and methionine metabolism; pyruvate metabolism; valine, leucine and isoleucine degradation; glycolysis/gluconeogenesis; 2-oxocarboxylic acid metabolism**	ribosome; protein processing in endoplasmic reticulum	PI3K-Akt signaling pathway; HIF-1 signaling pathway; ECM-receptor interaction	focal adhesion; lysosome	glucagon signaling pathway	Parkinson disease
**AEC**_**MV**_**vs. AEC**_**MV-free**_	cysteine and methionine metabolism; pyruvate metabolism; metabolic pathways; carbon metabolism; glycolysis/gluconeogenesis; glutathione metabolism; biosynthesis of amino acids; arginine biosynthesis; valine, leucine and isoleucine degradation; oxidative phosphorylation	**ribosome; protein processing in endoplasmic reticulum; proteasome**	–	lysosome	–	**Parkinson disease; Prion disease; Huntington disease; amyotrophic lateral sclerosis; Salmonella infection**
**AEC + LPS**_**MV**_**vs. AEC + LPS**_**TOT**_	*N/A*	*N/A*	*N/A*	*N/A*	*N/A*	*N/A*
**AEC + LPS**_**MV-free**_**vs. AEC + LPS**_**TOT**_	–	–	–	focal adhesion	–	–
**AEC + LPS**_**MV**_**vs. AEC + LPS**_**MV-free**_	**metabolic pathways**	–	–	–	–	–

DAPs were mapped to KEGG pathways and grouped according to the major functional categories defined by the KEGG database.

Boldface indicates pathways significantly enriched among higher-abundance proteins (log_2_FC > +1.5; *p* < 0.05), whereas regular type indicates pathways significantly enriched among lower-abundance proteins (log_2_FC < −0.67; *p* < 0.05). Italic type indicates pathways significantly enriched in both the higher- and lower-abundance protein subsets. Complete KEGG enrichment statistics, associated proteins, and protein-abundance rankings are provided in Table S2.

The MV fraction emerged as the principal reservoir of proteomic cargo, markedly surpassing its MV-free counterpart in protein content and reinforcing its central role in secretome functionality. Specifically, the MV fraction showed 546 higher abundant and 168 lower abundant proteins compared to the MV-free fraction (Figure 2B). KEGG pathway analysis of the higher abundant proteins identified a molecular signature characterized by mitochondria-associated metabolism (oxidative phosphorylation), as well as genetic information processes (ribosome, proteasome, protein processing in endoplasmic reticulum) and adaptive stress-response mechanisms (Salmonella infection, Parkinson disease, Alzheimer disease, Huntington disease, Prion disease, and amyotrophic lateral sclerosis) (Tables 1 and S2) thereby underscoring a prominent activation of adaptive stress-response mechanisms.

In contrast, lower abundant proteins were predominantly associated with pathways involved in glycolytic and anabolic metabolism (glycolysis/gluconeogenesis, pyruvate metabolism, amino acid biosynthesis, and glutathione metabolism) (Tables 1 and S2).

Several mitochondrial proteins, including VDAC1, TRAP1, ATP5PF, ATP5F1A, VDAC2, ATP5PB, and NDUFA4, were abundant in the MV fraction (Table S1). Among them, TRAP1 and PHB2 have been linked to immune and stress-response regulation. For instance, CCL20 is a chemokine involved in recruiting lymphocytes and dendritic cells to sites of inflammation. In addition, several proteins involved in cytokine and growth factor signaling pathways, including TGFBR3, TGFB1I1, MAP2K1, MAPK3, PRKAA1, and AKT1S1, were identified among the higher abundant proteins (Table S1). Of note, TGFBR3 serves as a co-receptor in the TGF-beta signaling pathway, which is pivotal in immune regulation and maintaining immune tolerance. Additionally, many components of the proteasome complex (PSMD1,2,3,7,5,11,12,13) play essential roles in antigen processing and presentation. The presence of these proteins suggests that MVs derived from AECs, especially under inflammatory conditions, carry a repertoire of proteins capable of modulating immune responses.

Together, these findings support the potential of AEC_MV_ as vehicles for delivering immunomodulatory signals, highlighting their prospective utility in developing therapeutic strategies aimed at immune suppression in inflammatory diseases.

Moving to the evaluation of the effects of LPS stimulation on the proteomic composition of the distinct secretome fractions, the most pronounced changes were once again observed within the AEC_MV_ fraction (Figure 2C). Specifically, LPS stimulation led to the increased abundance of 101 proteins (Figure 2D) enriched in KEGG pathways associated with metabolism. The top 5% of the proteins of this category showed enrichment of immune-related proteins, including CXCL1 and AREG (Figure 2C). While CXCL1 primarily functions as a chemoattractant driving leukocyte recruitment during inflammation.^41^ AREG has been increasingly implicated in tissue immunoregulation and repair.^16^^,^^33^^,^^34^^,^^35^^,^^36^ Moreover, AREG has been identified as a biologically active component of EV cargo communication.^42^ Notably, other abundant proteins in AEC + LPS_MV_ group showed immunomodulatory (PTX3, NHLRC2, and SGSH) and tissue-remodeling functions, including (MMP12, MMP3, MMP13, SERPINF1, FMOD, CTSL, and CTSC) (Table S2). Collectively, these findings indicate that inflammatory stimulation selectively remodels the molecular cargo of AEC_MV_, reflecting a targeted adaptive response to environmental cues. In contrast, LPS-induced changes in the AEC_TOT_ and AEC_MV-free_ fractions were less pronounced and more heterogeneous, supporting the notion that MVs represent the principal effector compartment of AEC paracrine signaling under inflammatory conditions. Within this vesicular fraction, AREG emerged among the most abundant immune-related proteins and, given its established role as a biologically active EV cargo component, represents a strong candidate mediator of MV-dependent immunomodulatory communication.

### Genetic silencing of AREG in AECs abrogates its cellular reservoir and MV-associated cargo

**Figure 3:**
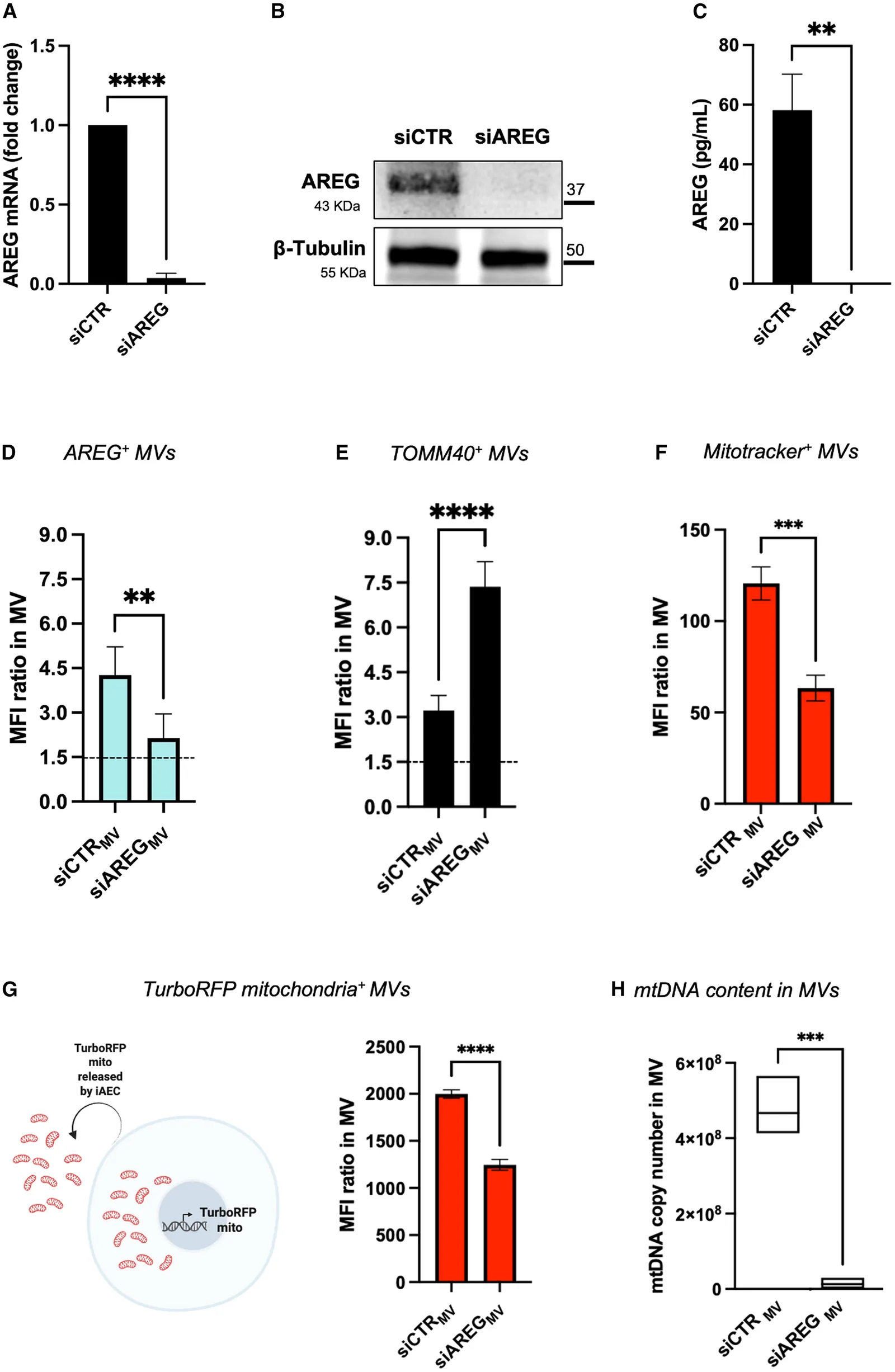
Genetic silencing of AREG in AECs abrogates its cellular reservoir and MV-associated cargo (A–C) Quantification of AREG expression following gene silencing in LPS-stimulated AECs, assessed by quantitative reverse-transcription PCR (RT-qPCR) for AREG mRNA levels (A), immunoblotting analysis of intracellular AREG protein (B), and ELISA measurement of secreted AREG in conditioned medium (C). (D) Flow cytometric quantification of AREG levels in MVs using an anti-AREG antibody. (E) Immunoblot analysis and densitometric quantification of TOMM40 enrichment in MVs following AREG silencing. (F) Flow cytometric quantification of mitochondrial labeling in MVs. (G) Flow cytometric analysis of MVs released by immortalized human AECs (iAECs) stably expressing mitochondria-targeted TurboRFP following treatment with control siRNA (siCTR) or AREG-targeting siRNA (siAREG). The schematic illustration was created with BioRender.com. (H) Mitochondrial DNA copy number in MVs derived from siCTR- and siAREG-treated AECs, quantified by real-time qPCR. Data are presented as mean ± SD from three biological independent experiments, with each biological replicate analyzed in at least three technical replicates. Comparisons between the two indicated groups were performed using an unpaired, two-tailed Student’s *t* test (A)–(H). ∗*p* < 0.05, ∗∗*p* < 0.01, ∗∗∗*p* < 0.001, ∗∗∗∗*p* < 0.0001. Only statistically significant differences are indicated.

To investigate the biological relevance of MV-associated AREG, we performed transient AREG gene knockdown in AEC exposed to LPS stimulation. AEC transfected with a non-targeting small interfering RNA (siRNA, siCTR) were used as a negative control. AREG downregulation was confirmed at intracellular levels, with significant reductions in mRNA (*p* < 0.0001; Figure 3A) and protein expression (Figure 3B) upon 48 h of transfection.

Consistently, secreted AREG levels analogously showed a marked depletion (*p* < 0.0001 vs. siCTR; Figure 3C). Notably, MVs derived from AREG-silenced AECs (siAREG_MV_) exhibited a 50% reduction in AREG positivity compared to control MVs, as demonstrated by fluorescence-activated cell sorting (FACS) analysis (*p* < 0.01, siAREG_MV_ vs. siCTR_MV_; Figure 3D), thereby confirming the efficacy of the silencing. Building on previous findings demonstrating that AEC immune fractions involve mitochondrial transfer via MVs,^15^ we examined whether AREG silencing affected mitochondrial content in MVs.

To assess the mitochondrial cargo of AEC-derived MVs, we adopted a multi-approach strategy combining the evaluation of the mitochondrial marker TOMM40, direct mitochondrial labeling with MitoTracker, mitochondrial-targeted TurboRFP tracing using an immortalized human AEC (iAEC) line stably expressing mitochondria-targeted turboRFP,^15^^,^^43^ and mtDNA quantification. While AREG silencing resulted in an increased abundance of TOMM40-positive MVs (*p* < 0.0001, siAREG_MV_ vs. siCTR_MV_; Figure 3E), the direct assessment of mitochondrial content by MitoTracker labeling (*p* < 0.001, siAREG_MV_ vs. siCTR_MV_ Figure 3F), together with complementary analyses performed using mitochondria-targeted TurboRFP-expressing iAECs (*p* < 0.0001, siAREG_MV_ vs. siCTR_MV_; Figure 3G) and mtDNA quantification (*p* < 0.001, siAREG_MV_ vs. siCTR_MV_; Figure 3H), demonstrated that AREG depletion was associated with a significant reduction in mitochondrial cargo within the MV fraction. These findings identify AREG as a novel modulator in mitochondrial MV assembly.

### AREG-silenced MVs show impaired internalization in immune cells

Building on our previous findings that AEC_MV_ can mediate mitochondrial transfer to immune cells, thereby promoting apoptotic signaling,^15^ we sought to determine the specific role of AREG depletion in this process. To verify whether AREG localized on the MV surface may interfere with mitochondrial internalization, we monitored mitochondrial cargo transfer into recipient immune cells (ovine PBMCs or human Jurkat T cells) mediated by MVs with or without AREG expression. The use of both species-specific (ovine-to-ovine and human-to-human) and interspecies (ovine-to-human) models was aimed to integrate advanced imaging and molecular quantification approaches.

**Figure 4:**
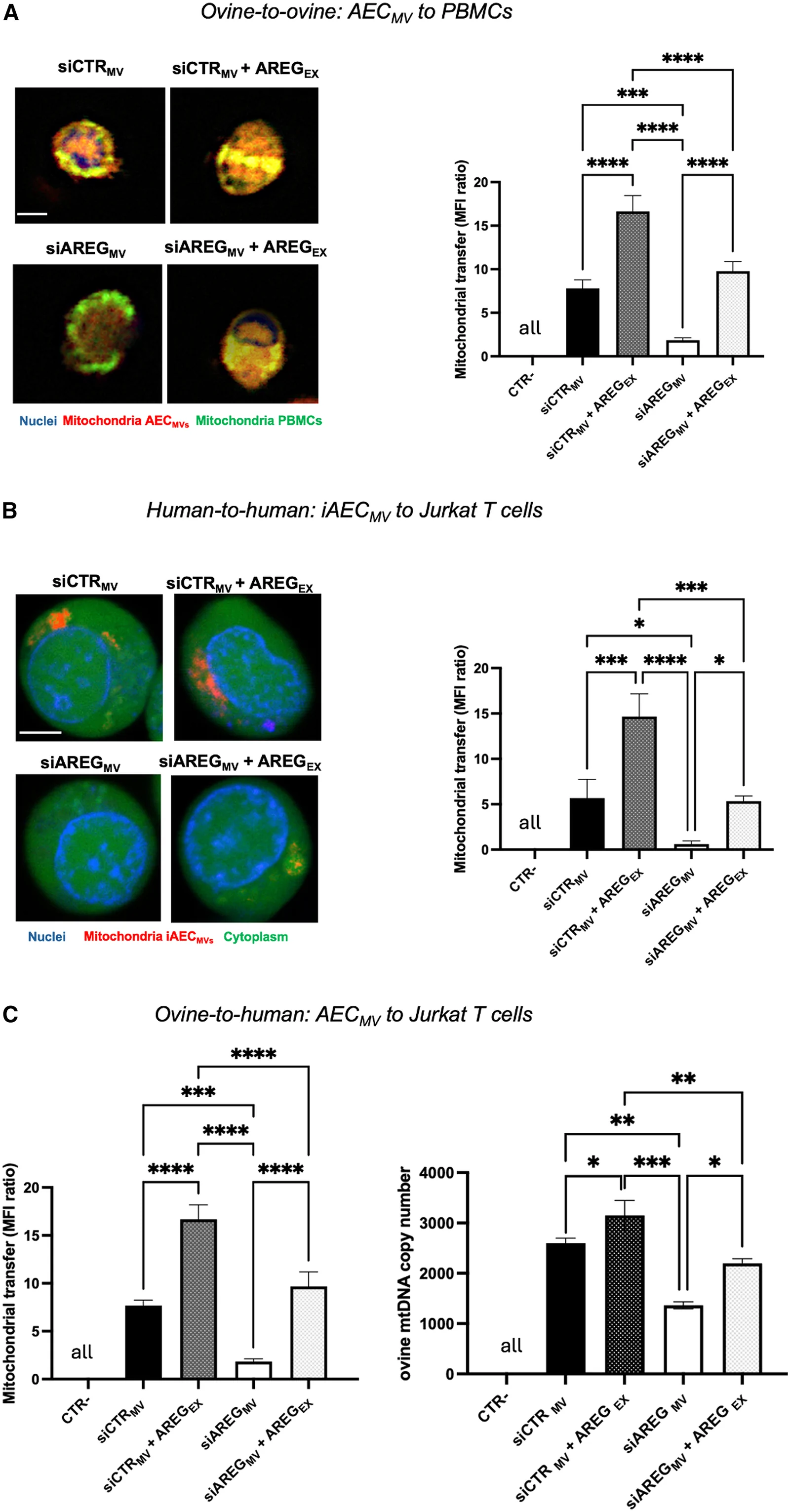
AREG-silenced MVs show impaired internalization in immune cells (A) Internalization of MitoTracker Red-labeled, MV-associated mitochondria by ovine peripheral blood mononuclear cells (PBMCs). PBMCs were incubated for 12 h with siCTR_MV_, siCTR_MV_ + AREG_EX,_ siAREG_MV_, or siAREG_MV_ + AREG_EX_ (AREG_EX_ denotes exogenously administered AREG). Representative confocal images (left) show nuclei stained with Hoechst 33342 (blue), endogenous PBMC mitochondria labeled with MitoTracker green (green), and MV-associated mitochondria labeled with MitoTracker Red (red). Mitochondrial uptake was quantified by flow cytometry (right). Scale bars, 5 μm. (B) Uptake of MV-associated mitochondria by human Jurkat T cells. MVs were derived from immortalized human AECs (iAECs) stably expressing mitochondria-targeted TurboRFP and subjected to the indicated AREG-modulating conditions. Representative confocal images (left) show nuclei stained with Hoechst 33342 (blue), cytoplasm labeled with Calcein AM (green), and TurboRFP-labeled mitochondria (red) after 12 h of incubation. Mitochondrial uptake was quantified by flow cytometry (right). Scale bars, 5 μm. (C) Interspecies mitochondrial transfer following incubation of human Jurkat T cells with MitoTracker Red-labeled MVs derived from ovine AECs. Mitochondrial uptake was quantified by flow cytometry (left), whereas the presence of ovine mtDNA in recipient cells was assessed by qPCR (right) to confirm cross-species transfer. Flow cytometry results are expressed as mean fluorescence intensity (MFI) ratios for cells positive for MitoTracker Red- or TurboRFP-labeled mitochondria. CTR indicates untreated PBMCs or Jurkat T cells. Data are presented as mean ± SD from three biological independent experiments, with each biological replicate analyzed in at least three technical replicates. Statistical significance was assessed by two-way ANOVA followed by Tukey’s multiple-comparisons test (A)–(C). ∗*p* < 0.05, ∗∗*p* < 0.01, ∗∗∗*p* < 0.001, ∗∗∗∗*p* < 0.0001. “All” indicates *p* < 0.05 versus all other experimental conditions. Only statistically significant comparisons are indicated.

In the species-specific setting, confocal microscopy (Figure 4A, left) revealed the internalization of red MitoTracker-labeled mitochondria from siCTR_MV_ into ovine PBMCs, co-localizing with endogenous mitochondria (green), as evidenced by the orange merged signal. Notably, this uptake was enhanced by exogenous AREG addition during incubation (siCTR_MV_ + AREG_EX_), while it was nearly abolished upon AREG knockdown (siAREG_MV_), and only partially rescued by re-supplementing AREG (siAREG_MV_ + AREG_EX_), indicating an AREG-dependent, dose, and spatial-responsive mechanism for mitochondrial transfer.

The negative influence of siAREG mitochondria internalization was quantified by flow cytometry analyses (Figure 4A, right) confirming the reduced PBMCs mitochondria uptake upon AREG silencing.

To exclude dye-dependent artifacts and further validate this phenotype, we used, in parallel, the tagged turboRFP iAEC. Upon confirming the silencing of AREG (Figure S2), we observed a consistent effect on human Jurkat cells: mitochondrial transfer was impaired by AREG depletion and partly rescued by AREG reintroduction, as shown by confocal imaging (Figure 4B, left) and flow cytometry (Figure 4B, right).

In the interspecies context, red MitoTracker-labeled MVs from ovine AECs were incubated with Jurkat cells for 12 h. Flow cytometry (Figure 4C, left) confirmed the previous pattern: reduced mitochondrial incorporation with AREG-deficient MVs. To quantify mitochondrial transfer, we also analyzed the ovine-transferred mtDNA copy number in human Jurkat cells (Figure 4C, right), as previously validated.^15^^,^^44^ The analysis confirmed significantly reduced mtDNA uptake in cells exposed to siAREG_MV_ vs. siCTR_MV_ (*p* < 0.01), and rescue with AREG reintroduction (*p* < 0.001, siAREG_MV_ + AREG_EX_ vs. siAREG_MV_; Figure 4C, right). Altogether, these results indicate that MV-associated AREG contributes to efficient mitochondrial transfer and uptake by immune recipient cells.

### AREG drives the immunosuppressive function of AEC_MV_ in immune cells

Following the confirmation of the AREG role in MV internalization into immune cells, finally, their immunomodulatory role was assessed. More in detail, MV derived from siAREG or siCTR cells were evaluated for their impact on activated immune cells such as ovine PBMCs and human Jurkat T cells.

**Figure 5:**
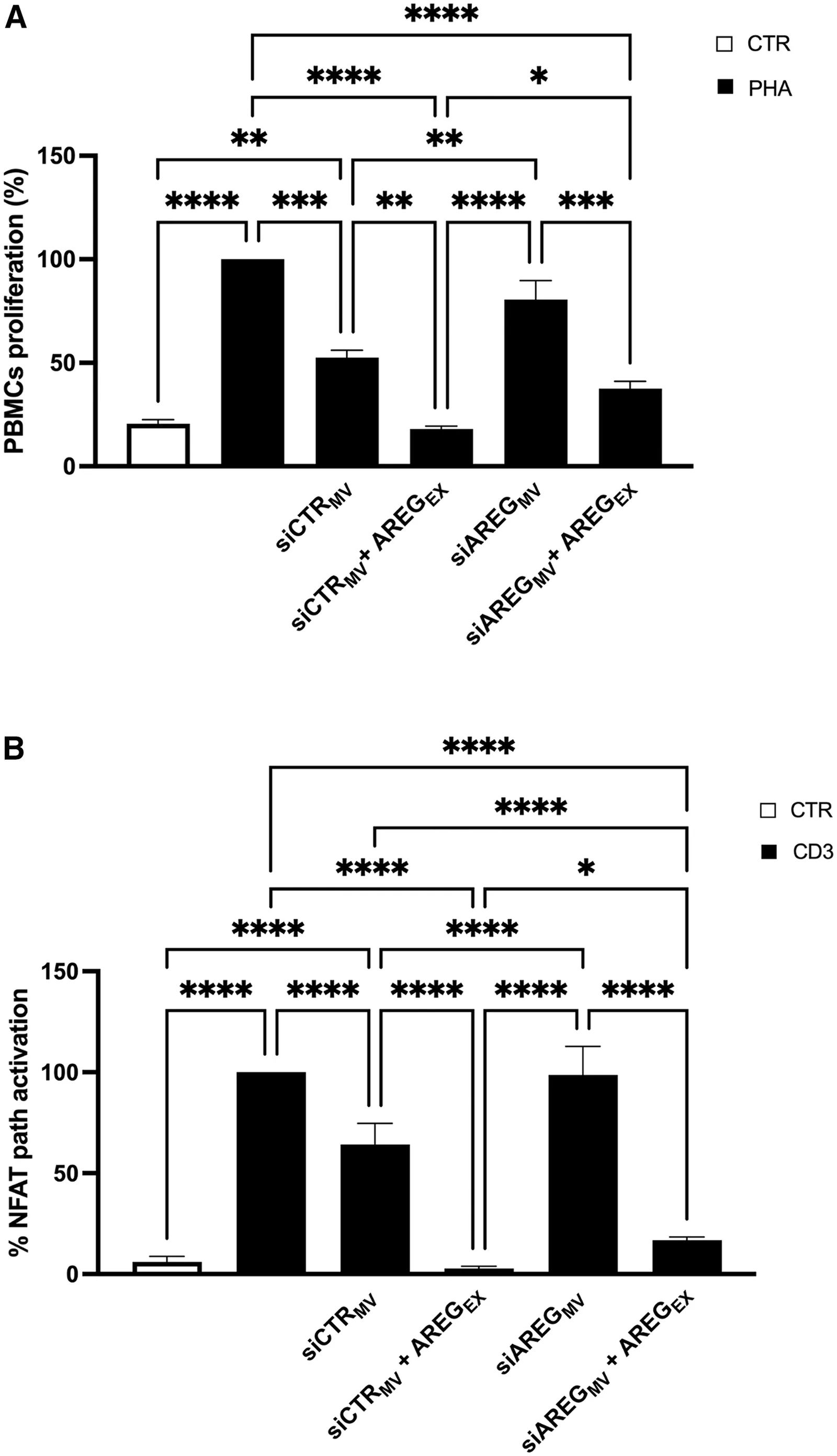
AREG drives the immunosuppressive function of AECMV in immune cells (A) Phytohemagglutinin (PHA)-activated PBMCs were incubated for 48 h with the indicated MV preparations (siCTR_MV_ ± AREG_EX_ and siAREG_MV_ ± AREG_EX_), and cell proliferation was assessed using the eFluor 450 cell proliferation dye. Values were normalized to those of PHA-stimulated PBMCs, which were defined as 100% proliferation. (B) CD3-stimulated Jurkat NFAT reporter cells were treated for 6 h with the same MV preparations, and NFAT activity was quantified to evaluate their inhibitory effects. Values were normalized to those of CD3-stimulated Jurkat cells, which were defined as 100% NFAT activation. Data are presented as mean ± SD from three biological independent experiments, with each biological replicate analyzed in at least three technical replicates. Statistical significance was assessed by two-way ANOVA followed by Tukey’s multiple-comparisons test (A)–(B). ∗*p* < 0.05, ∗∗*p* < 0.01, ∗∗∗*p* < 0.001, ∗∗∗∗*p* < 0.0001. Only statistically significant comparisons are indicated.

As shown in Figure 5A, MV derived from AECs transfected with a non-targeting siRNA confirmed their marked immunomodulatory effect, leading to a significant 47.5% reduction in cell proliferation under mitogenic stimulation (*p* < 0.01, siCTR_MV_ vs. phytohemagglutinin [PHA]). This effect was further enhanced by the exogenous supplementation of AREG, which potentiated siCTR_MV_ activity and lowered the proliferation rate to 25.5% (*p* < 0.05, siCTR_MV_ + AREG_EX_ vs. siCTR_MV_; Figure 5A). On the contrary, genetic ablation of AREG resulted in a dramatic loss of the immunomodulatory capacity of AEC_MV_ (*p* < 0.05, siCTR_MV_ vs. siAREG_MV_; Figure 5A) with PBMC that reached levels comparable to those observed under PHA-induced activation. Notably, the addition of exogenous AREG to siAREG effectively restored MVs modulatory function, leading to a 2.8-fold reduction in proliferation compared to the untreated silenced condition (*p* < 0.01, siAREG_MV_ + AREG_EX_ vs. siAREG_MV_; Figure 5A). However, AREG addition in this latter experiment did not restore the immunomodulatory influence recorded in the presence of siCTR + AREG thus indicating that the presence of AREG on the surface of MVs cannot be functionally replaced by protein exogenous supplementation (*p* < 0.05 siAREG + AREG vs*.* siCTR + AREG).

To further evaluate the impact of AREG silencing on the ability of MV cargo to modulate intracellular immune signaling pathways, the immunosuppressive effect of AEC_MV_ was assessed using interspecific Jurkat reporter cells responsive to CD3-induced NFAT activation.^15^^,^^45^^,^^46^^,^^47^^,^^48^ In detail, as reported in Figure 5B, MVs derived from siCTR AECs significantly reduced NFAT activation relative to CD3 stimulation (*p* < 0.0001, siCTR_MV_ vs. CD3), in line with the proliferation data. This effect was enhanced by exogenous AREG (*p* < 0.0001, siCTR_MV_ + AREG_EX_ vs. siCTR_MV_). In contrast, MVs from AREG-silenced AECs failed to suppress NFAT activation (*p* < 0.0001, siCTR_MV_ vs. siAREG_MV_; Figure 5B), reaching levels comparable to CD3-activated controls. Notably, the addition of AREG to siAREG_MV_ restored suppression of NFAT pathway activity (*p* < 0.0001, siAREG_MV_ + AREG_EX_ vs. siAREG_MV_; Figure 5B).

These findings underscore the pivotal role of AREG in conferring immunomodulatory properties to AEC_MV_, indicating that its presence within the MVs is critical for sustaining and enhancing their suppressive effects on immune cell proliferation and CD3-dependent NFAT path activation. Moreover, this effect appears to be mechanistically linked to MV uptake, as our previous work demonstrated that their immunosuppressive activity depends on internalization by immune cells, which was significantly impaired by treatment with pinocytosis inhibitors.^15^

## Discussion

Our comparative label-free proteomic analysis reveals that AEC_MV_ represent the dominant carriers of secretome proteins and display a markedly dynamic response to inflammatory stress.^49^^,^^50^ This finding supports previous reports that EVs from amniotic cells are selectively enriched in membrane-bound and secreted proteins, extracellular matrix components, and estrogen receptor (ER)-lumen residents.^51^^,^^52^ Additionally, it expands on these observations by demonstrating that inflammation acts as a licensing event that enhances the selectivity and functional sophistication of MV cargo. Our data demonstrate that LPS stimulation induces the accumulation of 101 differentially abundant proteins within the MV fraction, which converge functionally on metabolic reprogramming, revealing a refined and dynamic mechanism by which AEC_MV_ sculpt immune responses under inflammatory stress.

Among these, the significant upregulation of transcriptional regulators such as TGFBR3, and TGFB1I1, suggests a role for AEC_MV_ in shaping T helper cell polarization and promoting peripheral immune tolerance as proved for other amniotic cells.^53^^,^^54^ These proteins, previously identified within EVs, are known to be horizontally transferred to recipient immune cells, where they modulate transcriptional programs via STAT pathway activation.^55^ The concurrent enrichment of proteasomal subunits (e.g., PSMD proteins) supports mounting evidence that MV-associated proteasomes are catalytically active and contribute to MHC-I antigen processing, thereby extending the immune-sensing and antigen-presenting functions of AEC_MV_.^56^

The functional identity of these vesicles is highlighted by metabolic enzymes for glycolysis and cholesterol handling, showing AEC_MV_ as active immunometabolic units that can alter the bioenergetic state of macrophages and other immune effectors, a concept gaining traction in mesenchymal stromal cell EVs. Approximately 15% of MV-enriched proteins were mitochondrial, aligning with reports on vesicle-mediated mitochondrial transfer and its immunometabolic effects.^57^^,^^58^^,^^59^^,^^60^^,^^61^ Within this context, our data show that deficiency in AREG, an epidermal growth factor (EGFR) ligand with established immunomodulatory properties,^16^^,^^33^^,^^34^^,^^35^^,^^36^ decreases mitochondrial load of AEC_MV_ and reduces their uptake by immune cells. This suggests that efficient mitochondrial cargo delivery is jointly determined by cargo composition and MV surface-guidance cues, with AREG playing a prominent role. Interestingly, AREG expression was associated with enhanced MV internalization and lower mitochondrial cargo content, suggesting a potential role for AREG in modulating MV composition and transfer efficiency. This dual functionality highlights AREG as a critical molecular switch, orchestrating not only the compositional architecture but also the uptake and bioavailability of AEC_MV_ to recipient immune cells.

Expanding on these mechanistic insights, integrative proteomic profiling revealed additional immune-related cargo such as CXCL1, PTX3, SGSH, and NHLRC2, reinforcing the existence of a concerted enrichment strategy for immune-modulatory and stress-response pathways.^50^

Protein enrichment analyses confirmed the engagement of immunometabolic regulation, and proteasome-mediated antigen processing, all of which position AEC_MV_ as central players in the intercellular orchestration of immune responses. The enrichment of MVs in chemokines such as CCL20, which is known to generate haptotactic gradients that guide CCR6^+^ lymphocytes,^62^ might add a spatial layer to the immunoregulatory capabilities of these vesicles, suggesting that, in response to inflammatory cues, AECs exploit MV-based signaling not only to suppress immune overactivation but also to spatially coordinate immune cell positioning through chemotactic gradients.

Importantly, AREG emerged as a pivotal effector that can act not only in soluble form, as previously described,^16^ but also as a selectively packaged component of MVs, where it fulfills multiple roles. Genetic silencing of AREG in AECs resulted in MVs with significantly diminished ability to suppress PBMC proliferation and NFAT activation in Jurkat T cells, effects that were only partially restored by recombinant AREG supplementation.^16^^,^^33^^,^^34^^,^^35^^,^^36^ These observations support a model in which AREG is essential not only for modulating recipient immune cell activation via EGFR signaling but also for ensuring efficient MV uptake, possibly through receptor-mediated mechanisms.

Notably, this impaired immune modulation was accompanied by a defective mitochondrial transfer to immune cells, suggesting a potential association between these processes and a contributory role for AREG.

This resonates with studies on cancer-derived AREG-exosomes, whereas few as ∼24 molecules per vesicle are sufficient to elicit potent and specific EGFR responses.^42^ Interestingly, the presence of spliceosomal proteins (e.g., U2AF2, SF3B3, SF3B4, SNRPD1, SNRPA1, SNRNP200, SRSF1, SRSF3, SRSF6, and SRSF9), typically exported in EVs under stress to modulate RNA splicing and confer therapeutic resistance,^63^ in physiologic AEC_MV_ suggests an additional layer of regulatory influence whereby transcriptomic remodeling in recipient immune cells may contribute to long-term immunological quiescence.

Altogether, our findings advance the concept of AEC_MV_ as next-generation cell-free immunoregulatory tools. These vesicles orchestrate: (1) metabolic rewiring via mitochondrial transfer and glycolytic enzyme content; (2) direct immune suppression through AREG-dependent pathways; (3) chemotactic control via CCL20 gradients; and (4) modulation of antigen processing and RNA metabolism through proteasomal and spliceosomal cargo. Inflammatory cues, especially LPS, appear to tune the vesicular output of AECs to balance immune defense and prevent overactivation.

From a translational standpoint, AEC_MV_ enriched in AREG and other regulatory factors represent a promising avenue for cell-free therapeutic strategies in conditions marked by immune dysregulation and mitochondrial dysfunction—including graft-versus-host disease (GVHD),^64^ inflammatory bowel disease (IBD),^65^ neuroinflammatory conditions,^66^ and cancer-related immune dysfunctions.^67^ Unlike cell-based therapies, MVs offer advantages in safety, stability, and engineering potential. Future research should prioritize dissecting receptor-mediated uptake pathways, engineering AREG-enriched vesicles with enhanced targeting capabilities, and exploring synergistic interactions among co-packaged immunoregulatory molecules to maximize therapeutic efficacy.

### Limitations of the study

In this study, we provide evidence that MV-associated AREG shapes vesicle composition, mitochondrial transfer, and immunomodulatory activity in immune cells. Although AREG depletion markedly altered these readouts, the precise molecular basis of these effects remains unresolved. In particular, our experimental design does not allow us to disentangle potential direct roles of AREG in MV biogenesis, cargo selection, and interactions with recipient cells from broader consequences of AREG silencing on parental AECs and their secretome. Moreover, the reliance on siRNA-mediated knockdown and *in vitro* immune cell models may not fully capture the complexity of MV-immune cell crosstalk *in vivo*. Future studies employing complementary genetic strategies, in-depth receptor-ligand mapping, and disease-relevant preclinical models will be required to define the specific contribution of MV-associated AREG and to rigorously establish its translational relevance.

## Resource availability

### Lead contact

Requests for further information and resources should be directed to and will be fulfilled by the lead contact, Alessia Peserico (apeserico@unite.it).

### Materials availability

This study did not generate new unique reagents.

### Data and code availability

• Mass spectrometry proteomics data have been deposited at ProteomeXchange Consortium via the PRIDE partner repository as PXD074552 database identifier and are publicly available as of the date of publication.
• This paper does not report the original code.
• Any additional information required to reanalyze the data reported in this paper is available from the lead contact upon request.

## Acknowledgments

A.C.-V. acknowledges the European Union’s 10.13039/501100007601Horizon 2020 research and innovation program under the Marie Skłodowska-Curie grant agreement no. 955685 (www.p4fit.eu). A.C. has received funding from “Piano nazionale di ripresa e resilienza, fondo complementare Agribioserv,” Programma unitario di intervento per le aree del terremoto del 2009 e 2016, Misura B, Sub-misura B.4 - CUP: C43C21000150001- AARI00116. V.D.G. has received funding from the European Union-Next Generation EU, M4C1, project CUP: C46E23000120006, fellowship code: 39-411-A8-DOT13A8025-8428. H.A.S. acknowledges the 10.13039/501100005075UMCG research funds for financial support. The facilities and expertise of the Institute of Biotechnology and Professor Markku Varjosalo, at the University of Helsinki, are gratefully acknowledged for the proteomic analysis. Graphical abstract and some figures were created using BioRender.com.

## Author contributions

Conceptualization, B.B.; methodology, G.P. and A.C.-V.; formal analysis, G.P., A.C.-V., A.P., and A.C.; investigation, G.P., A.C.-V., A.P., A.C., V.D.G., M.T., O.D.G., and A.T.; writing – original draft, B.B., G.P., A.C.-V., and A.P.; writing – review and editing, G.P., A.C.-V., A.P., and A.C.; supervision, B.B.; H.A.S., T.M., and V.R.; project administration, B.B.; resources B.B., T.M., and C.T.; funding acquisition, B.B. and H.A.S.

## Declaration of interests

The authors declare no competing interests.

## STAR★Methods

### Key resources table

**Table:** 

REAGENT or RESOURCE	SOURCE	IDENTIFIER
**Antibodies**		

Anti-Tubulin Antibody	Cell Signaling Technology	Cat 2146; RRID: AB_2210545
Mouse monoclonal anti-CD3	Invitrogen, Thermo Fisher Scientific	Cat# 16-0037-81; RRID: AB_468854
Rabbit polyclonal anti-TOMM40	MyBiosource	Cat# MBS5400122; RRID: not available
Goat polyclonal anti-AREG	R&D Systems	Cat# AF262; RRID: AB_2243124
Mouse monoclonal anti-AREG	Invitrogen	Cat# 12-5370-42; RRID: AB_2716926

**Chemicals, peptides, and recombinant proteins**		

Tris(2-carboxyethyl)phosphine hydrochloride	Thermo Fisher Scientific	Cat# 20490
Iodoacetamide	Acros Organics	Cat# 122271000
Sequencing Grade Modified Trypsin	Promega	Cat# V5113
Trifluoroacetic acid (TFA)	VWR	Cat# 85049.051
Acetonitrile (HPLC grade)	VWR	Cat# 83640.320
HPLC grade water	Fisher Scientific	Cat# 10505904
TransIT-X2® Dynamic Delivery System	Mirus Bio LLC	Cat# MIR-6000
LPS	Sigma-Aldrich	Cat# L2637
PHA-L	Invitrogen	Cat# 00-4977-93
Penicillin-Streptomycin	Lonza	Cat# DE 17-602E
Amphotericin B	Euroclone	Cat# ECM0009D
L-Glutamine	Euroclone	Cat# ECB300D
P4	Sigma-Aldrich	Cat# P8783
Ficoll-Paque PLUS	Cytiva	Cat# GE17-1440-02
Fluoromount	Sigma-Aldrich	Cat# F4680
DPBS with Ca+2 and Mg+	Sigma-Aldrich	Cat# D8662
BSA	Sigma-Aldrich	Cat# A9418
Trypsin-EDTA 0.25%	Sigma-Aldrich	Cat# T4049
Quick Start™ Bradford 1x Dye Reagent	Bio-Rad Laboratories	Cat# 5000205
4X Laemmli Sample buffer	Bio-Rad Laboratories	Cat# 1610747
Phosphatase Inhibitor	SERVA Electrophoresis GmbH	Cat# 39055
Protease Inhibitor Cocktails	Sigma-Aldrich	Cat# P2714
Precast gel with a density gradient of 4–15%	Bio-Rad Laboratories	Cat# 4568083
Nitrocellulose membranes	Bio-Rad Laboratories	Cat# 1620145
Trans-Blot Turbo 5x Transfer Buffer	Bio-Rad Laboratories	Cat# 10026938
Every blot blocking solution	Bio-Rad Laboratories	Cat# 12010020
1X TBS 1% Casein Blocker	Bio-Rad Laboratories	Cat# 1610782
ClarityMax ECL reagent	Bio-Rad Laboratories	Cat# 1705062
Normocin™	InvivoGen	Cat#ANT-NR-2
Blasticidin	InvivoGen	Cat#ANT-BL-1
Zeocin®	InvivoGen	Cat#ANT-ZN-1
DMSO	Sigma-Aldrich	Cat# D8418
QUANTI-lucTM, Luciferase Detection Reagent	InvivoGen	Cat# REP-QLC4LG1
Mitotracker Red	Invitrogen	Cat# M7513
Mitotracker Green	Invitrogen	Cat# M7514
Hoechst 33342	Thermo Fisher Scientific	Cat# 62249
Calcein AM	Invitrogen	Cat# C3100MP

**Deposited data**		

Amniotic Epithelial Cell’s Secretome Proteomic Analysis	ProteomeXchange Consortium via the PRIDE partner repository	Database identifier: PXD074552

**Critical commercial assays**		

BioPureSPN PROTO 300 C18 Mini Columns	The Nest Group Inc.	Cat# HUM S18V
eBioscience™ Cell Proliferation Dye eFluor™ 450	Invitrogen	Cat# 65-0842-90
Sheep Amphiregulin (AREG) ELISA kit	MyBiosource	Cat# MBS044705
Human Amphiregulin ELISA kit	Invitrogen	Cat# EHAREG
Total RNA Purification Kit	Norgen Biotek Corp.	Cat# 17200
oligodT Primers	Bioline	Cat# BIO-38029
Tetro Reverse Transcriptase	Bioline	Cat# BIO-65050
Quick-DNA Miniprep Plus kit	Zymo Research Corporation	Cat# D4068
SYBR Lo-ROX kit	Bioline	Cat# BIO-94050

**Oligonucleotides**		

4 Custom ON-TARGETplus siRNA targeting ovine AREG	Dharmacon	oAREG 1: CCA UUA UAC UGC UGG AUU A oAREG 2: GAA GAA ACA GAA AGA GGA A oAREG 3: AGA AAC AGA AAG AGG AAA A oAREG 4: AAA GAA ACU UCG ACA AGA A
4 Custom ON-TARGETplus siRNA targeting human AREG	Dharmacon	hAREG 1: CCA CAG UGC UGA UGG AUU U hAREG 2: CAG GAA AUA UGA AGG AGA A hAREG 3: GCA AAU GUC AGC AAG AAU A hAREG 4: GAA GAA ACA GAA AGA AGA A
ON-TARGETplus Non-targeting Pool (NTC)	Dharmacon	Cat# D00181010 NTC_1:UGGUUUACAUGUCGACUAA NTC_2:UGGUUUACAUGUUUGUGUGA NTC_3:UGGUUUACAUGUUUUCUGA NTC_4:UGGUUUACAUGUUUUCCUA
Ovis Aries AREG forward primer	In house	5′-AAGGTGTCGCTGAAGAACGA-3′
Ovis Aries AREG reverse primer	In house	5′-TGATTCCGATCTGATACCCTGC-3′
Ovis Aries mtDNA forward primer	In house	5′-CTAGGCCTGTCCCTACTGGT-3′
Ovis Aries mtDNA reverse primer	In house	5′-GCAAGCTGTGAAGTGTGGTG-3′
Homo Sapiens AREG forward primer	In house	5′-GATACTCGGCTCAGGCCATT-3′
Homo Sapiens AREG reverse primer	In house	5′-ATGGTTCACGCTTCCCAGAG-3′
Homo Sapiens mtDNA forward primer	In house	5′-CACCCAAGAACAGGGTTTGT-3′
Homo Sapiens mtDNA reverse primer	In house	5′- TTAACAACATACCCATGGCCA-3′

**Experimental models**		

AEC	University of Teramo	N/A
iAEC	Nihon University School of Medicine	N/A
PBMCs	University of Teramo	N/A
Jurkat-LuciaTM NFAT cells	InvivoGen	Cat# jktl-nfat-cd28

**Software and algorithms**		

DIA-NN (v1.8.1)	Demichev Lab	https://github.com/vdemichev/DiaNN
timsControl	Bruker Daltonics	–
ImageJ 1.53k	NIH	https://imagej.nih.gov/ij/
GraphPad Prism 10	Graphpad	https://www.graphpad.com
Biorender	Biorender	https://www.biorender.com
NIS-Element software 4.40	Nikon	https://www.microscope.healthcare.nikon.com/products/software/nis-elements
CytExpert SRT	Beckman	https://www.beckman.it/flow-cytometry/research-flow-cytometers/cytoflex/software
ClueGO	Cytoscape	https://apps.cytoscape.org/apps/cluego

**Other**		

FBS	Gibco	Cat# 10270106
α MEM	Euroclone	Cat# ECM0850L
IMDM	Thermo Fisher Scientific	Cat# 12440053
DMEM	Euroclone	Cat# ECM0728L
C18 column (8 cm × 150 μm, 1.5 μm beads)	Evosep Biosystems	Cat# EV1109
96-well high-binding plate	Corning	Cat# 9018

### Experimental model and study participant details

Amniotic cells used in this study were isolated from adult pregnant sheep. No ethical approval was required, as amniotic membranes were obtained as waste material from animals slaughtered for food production in accordance with European Community Regulation (EC) No. 1069/2009. All procedures were performed in an institution authorized by the Italian Ministry of Health (approval number: ABP4169). Ovine PBMCs were isolated by peripheral blood collected at the slaughterhouse.

The reporter T cell line expressing an NFAT-inducible Lucia luciferase construct, Jurkat-Lucia™ NFAT cells, were purchrase form InvivoGen (#jktl-nfat-cd28; InvivoGen, Toulouse, France).

The iAEC cell line (immortalized human AEC expressing mitochondria-targeted turboRFP) was generously provided by the Toshio Miki Laboratory at the Nihon University School of Medicine (Tokyo, Japan). The identity of the iAECs cell line was not confirmed by short tandem repeat (STR) profiling. However, iAECs were routinely tested and confirmed to be negative for mycoplasma contamination.

### Method details

#### Isolation and culture of ovine amniotic-derived AEC

AEC were isolated from amniotic membranes (AM) collected from mid-gestation pregnant Appenninica ewes (fetal length: 25–30 cm),^11^ to prevent epithelial-mesenchymal transition (EMT) typically observed at term.^11^^,^^68^^,^^69^ The uterus wall was opened under sterile conditions, and the AM was mechanically separated from the chorion using a stereomicroscope and then cut into 2–3 cm fragments. AM pieces were washed in Dulbecco’s PBS (DPBS; #D8662; Sigma-Aldrich, St. Louis, MO, USA) and incubated under gentle agitation with 0.25% Trypsin-EDTA (#T4049; Sigma-Aldrich, St. Louis, MO, USA) at 38.5 °C for 40 min. Enzymatic digestion was stopped by adding 10% FBS (#10270106; Gibco, Thermo Fisher Scientific, Waltham, MA, USA), and the cell suspension was filtered through a 40 μm mesh. Cells were pelleted by centrifugation at 500 × g for 10 min, and viable cells were counted using the LUNA-II™ Automated Cell Counter (Logos Biosystems Inc., Gyeonggi-do, Korea) with trypan blue staining.

AEC were then seeded at 10^4^ cells/well in 6-well plates in α-MEM (#ECM0850L; Euroclone S.p.A., Milan, Italy) supplemented with 10% inactivated FBS (#10270106; Gibco, Thermo Fisher Scientific, Waltham, MA, USA), 1% L-Glutamine (#ECB300D; Euroclone S.p.A., Milan, Italy), 1% Amphotericin B (#EUM0009D; Euroclone S.p.A., Milan, Italy), and 1% penicillin/streptomycin (#DE17-602E; Lonza, Basel, Switzerland), referred to as complete medium, plus 25 μM Progesterone (P4; 4-pregnene-3,20-dione, #P8783; Sigma-Aldrich, St. Louis, MO, USA) to inhibit EMT.^70^^,^^71^ Cells were cultured at 38.5 °C with 5% CO_2_ until ∼70% confluence, then detached using 0.05% Trypsin-EDTA for subsequent experiments.

#### Isolation of ovine PBMCs

Ovine PBMCs were isolated by density gradient centrifugation from 16 mL of fresh peripheral blood. The gradient was prepared using 12 mL Ficoll-Paque™ PLUS (#GE17-1440-02; Cytiva, Marlborough, MA, USA), following the manufacturer’s protocol. Isolated cells were cryopreserved in liquid nitrogen until used in immunological assays.

#### Culture of jurkat reporter cells

Jurkat-Lucia™ NFAT cells were cultured in Iscove’s Modified Dulbecco’s Medium (IMDM; #12440053; Gibco, Thermo Fisher Scientific, Waltham, MA, USA), supplemented with 10% heat-inactivated FBS (#10270–106; Gibco, Thermo Fisher Scientific, Waltham, MA, USA), 100 μg/mL penicillin/streptomycin (#DE17-602E; Lonza, Basel, Switzerland), 100 μg/mL Normocin™ (#ANT-NR-2; InvivoGen, Toulouse, France), 5 μg/mL Blasticidin (#ANT-BL-1; InvivoGen, Toulouse, France), and 100 μg/mL Zeocin® (#ANT-ZN-1; InvivoGen, Toulouse, France). Cells were passaged every 2 days up to the third passage to maintain optimal density and cultured at 37 °C in a humidified 5% CO_2_ atmosphere.

#### iAEC cell culture

iAEC cell line was maintained in High Glucose DMEM (High Glucose DMEM; #ECM0728L; Euroclone S.p.A., Milan, Italy), supplemented with 10% heat-inactivated fetal bovine serum (FBS) (#10270106; Gibco, Thermo Fisher Scientific, Waltham, MA, USA), 1% L-Glutamine (#ECB300D; Euroclone S.p.A., Milan, Italy), and 1% penicillin-streptomycin (#DE17-602E; Lonza, Basel, Switzerland). This complete medium was used for routine culture, with cells being subcultured every 48 h at 37 °C in a humidified incubator with 5% CO_2_.

#### AEC/iAEC transient silencing of AREG and CM production

Transient gene depletion in AEC and iAEC was achieved using small interfering RNAs (siRNAs) at a final concentration of 50 nM. ON-TARGETplus siRNAs targeting AREG (#; Dharmacon, Horizon Discovery, Lafayette, CO, USA), and the corresponding Non-Targeting Control (NTC) siRNA (#FE5D0018101005; Dharmacon, Horizon Discovery, Lafayette, CO, USA) were used. Transfections were carried out using the TransIT-X2® Dynamic Delivery System (#MIR-6000; Mirus Bio LLC, Madison, WI, USA) according to the manufacturer’s instructions. To optimize transfection efficiency, cells were cultured until reaching 50–60% confluency with ≤2% non-viable cells. Transient gene silencing was then performed using the specific siRNAs. After 24 h, cells were preconditioned for 4 h in a serum-free medium (αMEM supplemented with 1% penicillin/streptomycin, 1% amphotericin B, and 1% L-glutamine). As previously described for AEC^15^^,^^16^^,^^71^^,^^72^ and other models,^73^ a subset of cells was treated with LPS (1 μg/mL; #L2637, Sigma-Aldrich, St. Louis, MO, USA) for 1 h. After treatment, all experimental groups (control and LPS-treated) were washed twice with serum-free medium and cultured for an additional 24 h in fresh serum-free medium containing siRNAs (a second round of transfection was performed during this period before CM collection). CM was harvested by centrifugation at 500 × g for 10 min, and supernatants were stored at −80 °C. Cell pellets were collected to verify the efficiency of gene silencing via quantitative real-time PCR and Western blot analysis.

• 4 custom ON-TARGETplus siRNA targeting ovine AREG (Dharmacon, Horizon Discovery, Lafayette, CO, USA) used as a pool:
  ○ oAREG 1: CCA UUA UAC UGC UGG AUU A
  ○ oAREG 2: GAA GAA ACA GAA AGA GGA A
  ○ oAREG 3: AGA AAC AGA AAG AGG AAA A
  ○ oAREG 4: AAA GAA ACU UCG ACA AGA A
• 4 Custom ON-TARGETplus targeting human AREG (Dharmacon, Horizon Discovery, Lafayette, CO, USA) used as a pool:
  ○ hAREG 1: CCA CAG UGC UGA UGG AUU U
  ○ hAREG 2: CAG GAA AUA UGA AGG AGA A
  ○ hAREG 3: GCA AAU GUC AGC AAG AAU A
  ○ hAREG 4: GAA GAA ACA GAA AGA AGA A
• ON-TARGETplus Non-targeting Pool (NTC) for both ovine and human (#D00181010; Dharmacon, Horizon Discovery, Lafayette, CO, USA):
  ○ NTC_1: UGGUUUACAUGUCGACUAA;
  ○ NTC_2: UGGUUUACAUGUUUGUGUGA;
  ○ NTC_3: UGGUUUACAUGUUUUCUGA;
  ○ NTC_4: UGGUUUACAUGUUUUCCUA

#### MV isolation

MV fractions were isolated from collected CM by ultracentrifugation at 25,000 × g for 5 min at 4 °C using an Optima™ XPN-90 ultracentrifuge (Beckman Coulter Life Sciences, Brea, CA, USA), as previously described.^15^ The supernatant was discarded, and the MV-containing pellet was resuspended in a serum-free culture medium for downstream applications. To ensure consistency of MV isolation, preparations were routinely assessed according to the characterization criteria previously established for this protocol,^15^ including particle size distribution by TRPS and the detection of vesicular (e.g., CD63) and organelle-associated markers (e.g., CNX, TOMM40, HSP60), thereby confirming enrichment of the recovered fraction in microvesicles.

#### Sample preparation for proteomics analysis

For proteomics characterization, total CM was ultracentrifuged at 25,000 × g for 5 min at 4 °C using an Optima™ XPN-90 ultracentrifuge (Beckman Coulter Life Sciences, Brea, CA, USA), as previously described.^15^The The resulting supernatant was collected as the MV-free fraction, while the pellet was defined as the MV fraction. The MV pellet was gently resuspended in a small volume of serum-free medium. Proteins from total CM, MV-free and MV fractions were precipitated using trichloroacetic acid (TCA). Briefly, 100% (w/v) TCA was added to each sample to achieve a final concentration of 20% (w/v). Samples were incubated on ice for at least 1 h and subsequently centrifuged at 16,000 × g for 10 min at 4°C. The resulting protein pellets were washed three times with ice-cold 0.01 M HCl in 90% acetone and then air-dried. Dried protein pellets were resuspended in 100 mM ammonium bicarbonate (NH4HCO3) containing 8 M urea.

15 μg of total protein from each sample was taken for proteomics analysis. The urea concentration was diluted to 1 M with 100 mM NH4HCO3. Then the sample types were reduced with 5 mM Tris(2-carboxyethyl)phosphine hydrochloride (#20490; Thermo Fisher Scientific, Waltham, MA, USA), alkylated with 10 mM iodoacetamide (#122271000; Acros Organics, Geel, Belgium) at room temperature, and trypsin-digested at 37°C for 16 h using Seguencing Grade Modified Trypsin (#V5113; Promega Corporation, Madison, WI, USA). After digestion, samples were acidified with 10% trifluoroacetic acid (TFA, #85049.051; VWR International, Radnor, PA, USA) and desalted with BioPureSPN PROTO 300 C18 Mini columns (#HUM S18V; The Nest Group Inc., Southborough, MA, USA) according to the manufacturer’s instructions. After desalting, the samples were dried in a centrifuge concentrator (Concentrator Plus, Eppendorf SE, Hamburg, Germany). The dried peptides were reconstituted in 30 μL buffer A (0.1% (vol/vol) TFA, 1% (vol/vol) acetonitrile (#83640.320; VWR International, Radnor, PA, USA) in HPLC grade water (#10505904; Fisher Scientific UK Ltd., Loughborough, UK).

For the DIA analysis of samples, the resuspended peptides were further diluted to 7.5 ng/μL in buffer A1 (1% formic acid in HPLC water). 20 μL was loaded into an Evotip (Evosep Biosystems, Odense, Denmark) following the manufacturer’s instructions to achieve 150 ng of peptide load for the analysis.

#### Mass spectrometry and data analysis

The desalted samples were analyzed using the Evosep One liquid chromatography system (Evosep Biosystems, Odense, Denmark) coupled to a hybrid trapped ion mobility quadrupole TOF mass spectrometer (Bruker timsTOF Pro, Bruker Daltonics GmbH & Co. KG, Bremen, Germany)^74^ via a CaptiveSpray nano-electrospray ion source (Bruker Daltonics GmbH & Co. KG, Bremen, Germany). An 8 cm × 150 μm column with 1.5 μm C18 beads (EV1109, Evosep Biosystems, Odense, Denmark) was used for peptide separation with the 60 samples per day method (21 min gradient time). Mobile phases A and B were 0.1% formic acid in water and 0.1% formic acid in acetonitrile, respectively. The MS analysis was performed in the positive-ion mode with the dia-PASEF method^75^ with sample-optimized data-independent analysis (dia) scan parameters. We performed DDA in PASEF mode from a pooled sample to be able to adjust dia-PASEF parameters optimally to these specific samples. To perform sample-specific dia-PASEF parameter adjustment, the default dia-short-gradient acquisition methods were adjusted based on the sample-specific DDA-PASEF run with the software “timsControl” (Bruker Daltonics GmbH & Co. KG, Bremen, Germany). The following parameters were modified for each sample type: m/z range; 430.0–1337.0, mass steps per cycle; 37 mean cycle time; 1.91 s. The ion mobility windows were set to best match the ion cloud density from the sample type-specific DDA runs.

To analyze diaPASEF data, the raw data (.d) were processed with DIA-NN v18.0^76^^,^^77^ utilizing a spectral library generated from the UniProt sheep proteome (Uniprot Ovis Aries Reference Proteome, downloaded 06.03.2024 as a FASTA file, 49510 proteins). During library generation following settings were used, fixed modifications: carbamidomethyl (C); variable modifications: acetyl (protein N-term), oxidation (M); enzyme: Trypsin/P; maximum missed cleavages:1; mass accuracy fixed to 1.5e−05 (MS2) and 1.5e−05 (MS1); Fragment m/z set to 100–1600; peptide length set to 7–50; precursor m/z set to 200–1700; Precursor changes set to 2–4; protein inference not performed. All other settings were left to default.

The mass spectrometry proteomics data have been deposited to the ProteomeXchange Consortium via the Proteomics Identifications Database (PRIDE) partner repository with the database identifier: PXD074552.

Ontology analysis was performed by using the Cytoscape ClueGO plugin^78^ to seek KEGG pathways enrichment of the collected proteomes from the different secretome fractions. The reference database used for pathway analysis was Ovis aries (Taxid: 9940).

#### Flow cytometry

For MV characterization, TOMM40 (1:100; #MBS5400122, MyBioSource, San Diego, CA, USA) and AREG (5 μL (0.25 μg)/test; #12-5370-42; Invitrogen, Thermo Fisher Scientific, Waltham, MA, USA) antibodies were employed. In addition, MitoTracker® Red CM-H2XRos (500 nM; #M7513; Invitrogen, Thermo Fisher Scientific, Waltham, MA, USA) and eBioscience™ Cell Proliferation Dye eFluor™ 450 (10 μM; #65–0842; Invitrogen, Thermo Fisher Scientific, Waltham, MA, USA) were employed. Samples were thoroughly washed with Dulbecco’s PBS (DPBS; #D8662; Sigma-Aldrich, St. Louis, MO, USA) and resuspended in DPBS supplemented with 0.5% BSA (#A3059; Sigma-Aldrich, St. Louis, MO, USA). Flow cytometry was performed using a CytoFLEX SRT system (Beckman Coulter, Brea, CA, USA), acquiring 10,000 events per sample. Excitation was carried out with 405 nm and 561 nm lasers, and fluorescence was detected in the V450 and Y585 channels using a standard logarithmic scale. Data were analyzed and visualized with CytExpert SRT software (version 1.2.10004; Beckman Coulter, Brea, CA, USA).

#### Western blotting

Total protein was extracted from each sample using RIPA lysis buffer (#R0278; Sigma-Aldrich, St. Louis, MO, USA) supplemented with Phosphatase Inhibitor (#39055; SERVA Electrophoresis GmbH, Heidelberg, Germany) and Protease Inhibitor Cocktail (#P2714; Sigma-Aldrich, St. Louis, MO, USA), diluted according to the manufacturer’s instructions. Samples were incubated on ice for 30 min and centrifuged at 12,000 × g for 12 min at 4 °C. The supernatant was collected, and 5 μL was used to determine protein concentration using Quick Start™ Bradford 1x Dye Reagent (#5000205; Bio-Rad Laboratories, Milan, Italy). Proteins were separated on 4–15% gradient precast gels (#4568083; Mini-PROTEAN®, Bio-Rad Laboratories, Milan, Italy) and transferred onto nitrocellulose membranes (#1620145; Bio-Rad Laboratories, Milan, Italy) using the Trans-Blot® Turbo Transfer System and 5× Transfer Buffer (#10026938; Bio-Rad Laboratories, Milan, Italy). Membranes were blocked with EveryBlot™ Blocking Buffer (#12010020; Bio-Rad Laboratories, Milan, Italy) for 5 min. Primary antibody against AREG (1:200; #AF262; R&D Systems, Inc., Minneapolis, MN, USA) and beta-Tubulin (1:2000; #2146; Cell Signaling Technology) was diluted in 1× TBS containing 1% Casein Blocker (#1610782; Bio-Rad Laboratories, Milan, Italy) and incubated overnight at 4 °C. The HRP-conjugated anti-goat secondary antibody (1:5000; #SC-2354; Santa Cruz Biotechnology, Inc., Dallas, TX, USA) was diluted in the same buffer and incubated for 1 h at room temperature. Detection was performed using Clarity Max™ ECL Substrate (#1705062; Bio-Rad Laboratories, Milan, Italy), and signals were acquired with the ChemiDoc™ MP Imaging System (Bio-Rad Laboratories, Milan, Italy). Densitometric analysis was carried out using ImageJ software (v1.53k; National Institutes of Health, Bethesda, MD, USA), and protein expression was normalized to total protein content using Stain-Free™ technology (Bio-Rad Laboratories, Milan, Italy).

#### ELISA assay

The CM were analyzed for AREG concentration using the “Sheep Amphiregulin (AREG) ELISA kit” (#MBS044705; MyBioSource, Southern California, San Diego, USA) and the “Human Amphiregulin ELISA kit” (#EHAREG; Invitrogen, Thermo Fisher Scientific, Waltham, MA, USA), following the manufacturers’ instructions.

#### Immunofluorescence

Mitochondria from PBMCs and MVs were stained with MitoTracker® Green FM (200 nM; #M7514; Invitrogen, Thermo Fisher Scientific, Waltham, MA, USA) and MitoTracker® Red CM-H2XRos (500 nM; #M7513; Invitrogen, Thermo Fisher Scientific, Waltham, MA, USA), according to the manufacturer’s instructions. For MV staining, samples were incubated with MitoTracker® Red for 30 min at 37 °C, centrifuged at 25,000 × g for 5 min, washed twice with cell culture medium (centrifugation repeated after each wash), and resuspended in medium or distilled water for subsequent use on PBMCs and Jurkat reporter cells. Cells were then exposed to stained AEC ± LPSMV for 12 h. Analyses were performed using flow cytometry or confocal microscopy. For confocal imaging, nuclei of PBMCs and Jurkat cells were counterstained with Hoechst 33342 (1:1000 in DPBS; #62249; Thermo Fisher Scientific, Waltham, MA, USA) for 10 min at room temperature. Jurkat cytoplasm was stained with Calcein AM (1:1000 in DPBS; #C3100MP; Invitrogen, Thermo Fisher Scientific, Waltham, MA, USA) for 30 min at room temperature.

#### RNA extraction

Total RNA from AECs was extracted using the Total RNA Purification Kit (#17200; Norgen Biotek Corp., Thorold, ON, Canada), following the manufacturer’s instructions. Subsequently, cDNA synthesis was performed starting from 1 μg of total RNA per sample using oligodT Primers (#BIO-38029; Bioline, Cincinnati, OH, USA) and Tetro Reverse Transcriptase (#BIO-65050; Bioline, Cincinnati, OH, USA), according to the manufacturer’s protocols.

#### DNA extraction

To assess the interspecific transfer of ovine MVs, DNA was extracted 12 h post-treatment from pooled samples of 1 × 10^6^ Jurkat reporter cells per condition, as well as from 1 mL of AEC_MV_ (positive control). Extraction was done using the Quick-DNA Miniprep Plus kit (#D4068; Zymo Research, Irvine, CA, USA) according to the manufacturer’s protocol. DNA concentration and purity were determined spectrophotometrically using a NanoDrop® 2000c (Thermo Fisher Scientific, Waltham, MA, USA), measuring absorbance at 230, 260, and 280 nm with 1 μL of sample.

#### Real-time qPCR

To quantify AREG in AEC and ovine mitochondrial DNA (mtDNA) transferred via MV-mediated mitochondrial uptake in Jurkat reporter cells, Real-Time qPCR was performed in triplicate using the SensiFAST SYBR Lo-ROX kit (#BIO-94050; Bioline, Cincinnati, OH, USA) on a QuantStudio™ 3 system (Applied Biosystems, Thermo Fisher Scientific, Waltham, MA, USA), following the manufacturer’s protocol. The thermal profile included an initial denaturation at 95 °C for 3 min, followed by 40 cycles at 95 °C for 5 s and 60 °C for 30 s. Primers (0.5 pmol/μL) specific to ovine mtDNA were designed using NCBI Primer-BLAST and validated via melting curve analysis. The mtDNA copy number was calculated as described by Takeda et al.^44^

• AREG primer sequences (Ovis Aries): forward 5′- AAGGTGTCGCTGAAGAACGA-3′ (Tm: 59.61°C) and reverse 5′-TGATTCCGATCTGATACCCTGC -3′ (Tm: 59.70°C).
• mtDNA primer sequences (Ovis Aries): forward 5′-CTAGGCCTGTCCCTACTGGT-3′ (Tm: 60.03 °C) and reverse 5′-GCAAGCTGTGAAGTGTGGTG-3′ (Tm: 59.97 °C).
• mtDNA primer sequences (Homo Sapiens): forward 5′- CACCCAAGAACAGGGTTTGT-3 (Tm: 63.0 °C) and reverse 5′- TTAACAACATACCCATGGCCA-3’ (Tm: 62.0 °C).
• AREG primer sequences (Homo Sapiens): forward 5′- GATACTCGGCTCAGGCCATT 3′ (Tm: 59.61 °C) and reverse 5′- ATGGTTCACGCTTCCCAGAG -3′ (Tm: 60.04°C).

#### PBMCs activation test

The immunomodulatory activity of MV fractions was evaluated using a PBMC activation assay. PBMCs (2 × 10^5^ cells/well) were seeded in 96-well plates, stimulated with 10 μg/mL PHA-L (#00-4977-93; Invitrogen, Thermo Fisher Scientific, Waltham, MA, USA), and cultured for 48 h with or without AEC ± LPS_MV_ fractions. Cell proliferation was assessed using eBioscience™ Cell Proliferation Dye eFluor™ 450 (10 μM; #65–0842, Invitrogen, Thermo Fisher Scientific, Waltham, MA, USA), following the manufacturer’s instructions. Data were normalized to PHA-stimulated controls.

#### NFAT pathway activation test

Jurkat-Lucia™ NFAT reporter cells were used to assess the effects of different MV fractions on intracellular immune signaling pathways related to proliferation. Cells, amplified for three passages, were seeded at 3 × 10^5^ cells/well in a 96-well high-binding plate (#9018; Corning, NY, USA) pre-coated with CD3 (2 μg/mL, #16-0037-81; Invitrogen, Thermo Fisher Scientific, Waltham, MA, USA). Cultures were maintained for 6 h with or without AEC ± LPS_MV_ fractions. Luminescence was measured following QUANTI-Luc™ reagent addition (#REP-QLC4LG1; InvivoGen, Toulouse, France) using an EnSpire® Multimode Plate Reader (PerkinElmer, Waltham, MA, USA). Data were normalized to CD3-stimulated controls.

#### Functional annotation and KEGG pathway analysis

High-abundant and low-abundant proteins identified in each secretome fraction were analyzed independently for Kyoto Encyclopedia of Genes and Genomes (KEGG) pathway enrichment. Significantly enriched pathways were retrieved and classified according to the first-level KEGG BRITE hierarchy (https://www.genome.jp/kegg/pathway.html). Pathways were grouped into the major KEGG functional categories, and the frequency of enriched pathways within each category was calculated to provide an overview of the biological functions represented in each protein dataset.

### Quantification and statistical analysis

A minimum of three biological replicates were used for AEC (fetal) and PBMC (animal) samples to assess inter-experimental variability. Each experiment was repeated three times to evaluate intra-experimental consistency. Sample sizes (n) and statistical significance are reported in the figure legends. Data are shown as mean ± S.D. Normality was tested using the D'Agostino and Pearson test. two-way ANOVA and appropriate unpaired t-tests were applied to normally distributed data, followed by Tukey’s post hoc test (GraphPad Prism 10, San Diego, CA, USA). Significance was set at *p* ≤ 0.05.

For proteomic data, the input file for further DIA data analysis was the DIA-NN Report.pg_matrix. Data preprocessing was performed using a custom in-house R script. Raw intensity values were log_2_-transformed and median-normalized. Afterward, missing values were imputed using Random Forest imputation.^79^ For sample group comparison, *p*-values were calculated with the Student’s *t* test using the Python package scipy^80^ and adjusted using the Benjamini-Hochberg method via the statsmodels package.^81^ Volcano plots were generated with GraphPad Prism using a *p*-value threshold of 0.05 and log2 intensity fold change thresholds > +1.5 (most abundant proteins) and < −0.67 (less abundant proteins).

## Supplemental information

Document S1. Figures S1 and S2

Table S1. Proteomic dataset of UniProt-referenced Ovis aries proteins identified in this study, related to Figure 1 The table reports the expression values for each protein across all experimental samples, including the values obtained from each of the three biological replicates. Missing values were imputed using the random forest method. Each protein entry is annotated with its UniProt accession number, protein name, and corresponding gene symbol. For comparisons between sample groups, *p* values were calculated using Student’s *t* test implemented in the Python SciPy package and adjusted for multiple comparisons using the Benjamini-Hochberg method implemented in the stats models package

Table S2. KEGG pathway enrichment analysis, related to Table 1 Tables report KEGG pathway enrichment analyses performed on proteins with increased abundance (log_2_FC > +1.5, *p* < 0.05) and decreased abundance (log_2_FC < −0.67, *p* < 0.05) identified in the indicated secretome fraction comparisons. Functional enrichment analysis was performed using the Search Tool for the Retrieval of Interacting Genes/Proteins) STRING database (v.12.0) for Ovis aries, with the Ovis aries proteome used as the statistical background. Enrichment *p* values were corrected for multiple testing using the Benjamini-Hochberg method, and KEGG pathways with a false discovery rate (FDR) < 0.05 were considered significantly enriched. For each pathway, the tables report the observed protein count, background protein count, enrichment strength, signal, FDR, and matching proteins. Proteins are ranked according to their relative abundance, with the top 5% most abundant proteins highlighted in pink
