# Supplementary material for: Amphiregulin shapes mitochondrial delivery and immune modulation by microvescicles
Source: iScience. 2026 Sep 3;29(9):117339. doi: 10.1016/j.isci.2026.117339 (PMC13571665; doi:10.1016/j.isci.2026.117339)
Supplement: Supplementary file 1 [file mmc1.pdf]

iScience, Volume *29*

Supplemental information

Amphiregulin shapes mitochondrial delivery and immune modulation by microvescicles

Giuseppe Prencipe, Adrián Cerveró-Varona, Alessia Peserico, Angelo Canciello, Verdiana Di Giulio, Hélder A. Santos, Antti Tuhkala, Chika Takano, Toshio Miki, Valentina Russo, Maura Turriani, Oriana Di Giacinto, and Barbara Barboni

iScience, Volume ■■

Supplemental information

Amphiregulin shapes mitochondrial delivery

and immune modulation by microvescicles

Giuseppe Prencipe, Adrián Cerveró-Varona, Alessia Peserico, Angelo Canciello, Verdiana Di Giulio, Hélder A. Santos, Antti Tuhkala, Chika Takano, Toshio Miki, Valentina Russo, Maura Turriani, Oriana Di Giacinto, and Barbara Barboni

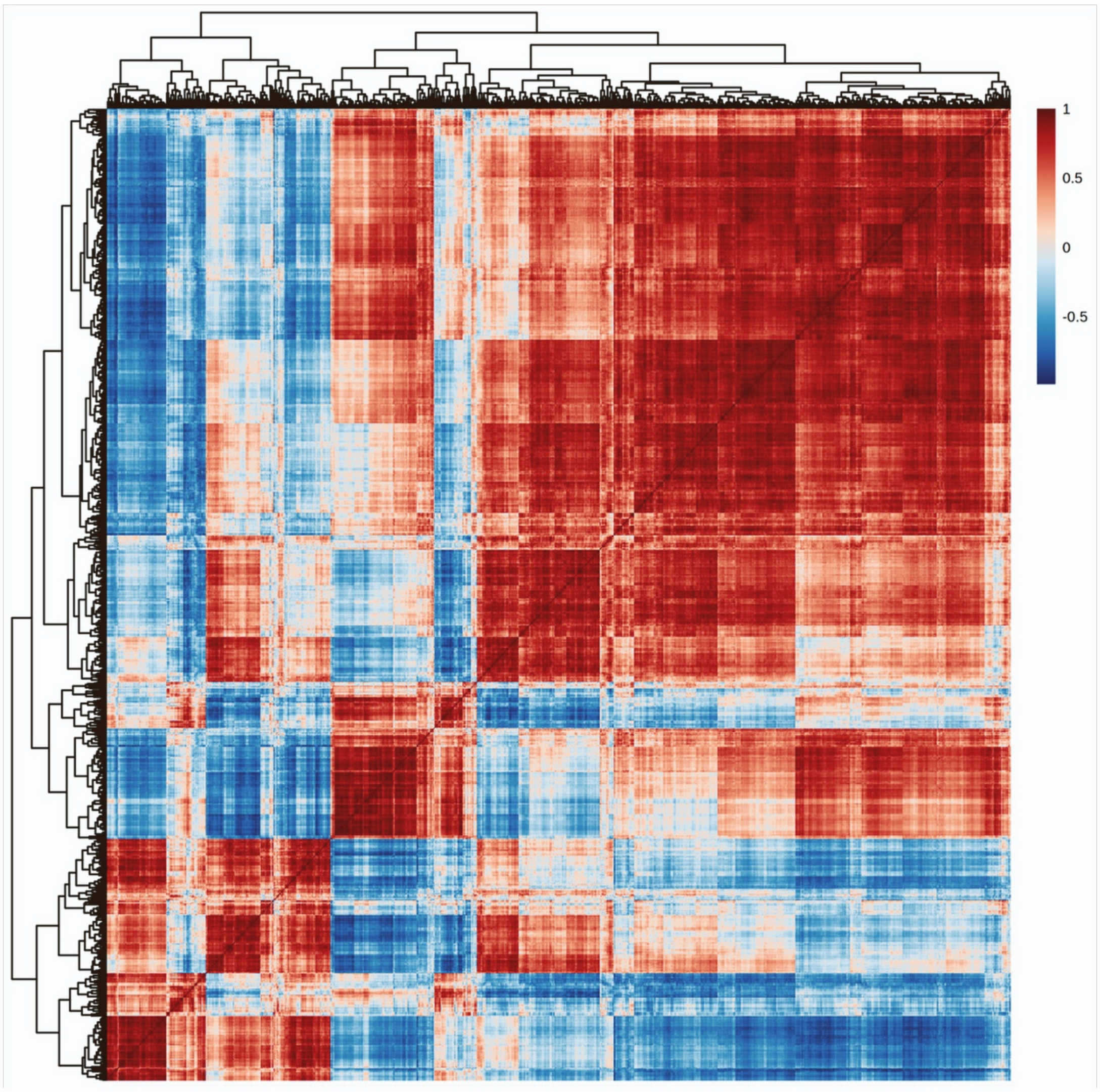

Figure S1. Pairwise correlation matrix of protein abundance profiles

Related to Figure 1. Pearson's correlation coefficients were calculated from protein abundance values across all experimental samples. Rows and columns represent individual proteins, and colors indicate the direction and strength of the correlations, ranging from negative (blue) to positive (red) correlations. Proteins were ordered by hierarchical clustering. The analysis was performed using individual protein abundance values from three independent biological experiments.

Figure S2

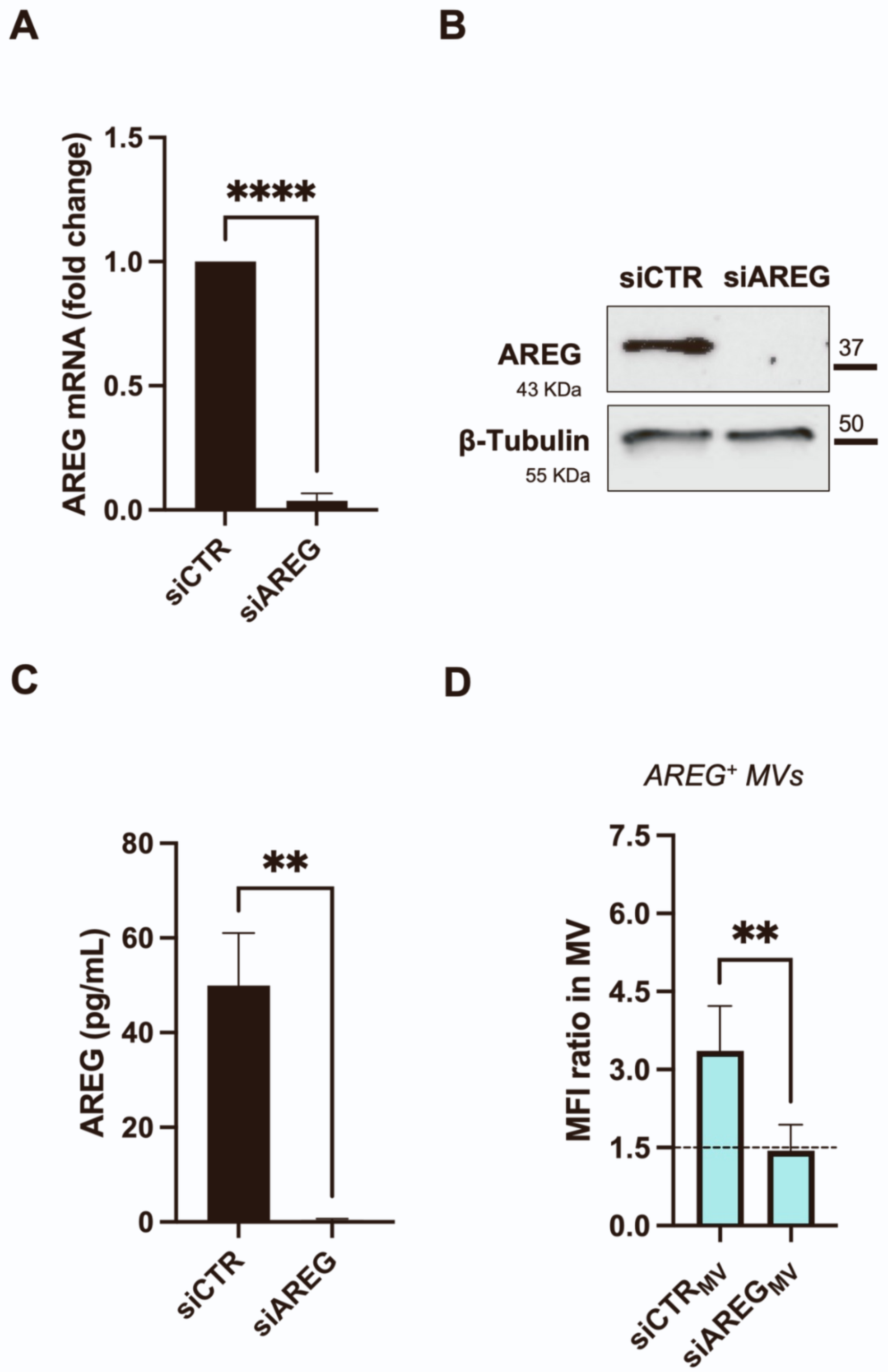

Figure S2. Genetic Silencing of AREG in iAECs Abrogates its Cellular Reservoir and MV-Associated Cargo.

Related to Figure 4. (A-C) Quantification of AREG expression following gene silencing in LPS-stimulated iAECs, measured by qPCR for mRNA levels (A), immunoblotting for intracellular AREG (B), and ELISA for secreted AREG in conditioned medium (C). (D) Flow cytometry analysis of AREG levels in MVs, using an anti-AREG antibody. Data are presented as mean ± SD from three biological independent experiments, with each biological replicate analyzed in at least three technical replicates. Comparisons between the two indicated groups were performed using an unpaired, two-tailed Student's t test (A–D). $^{**}p < 0.01$ and $^{****}p < 0.0001$. Only statistically significant differences are indicated.
